# Triple-Decker Hexaazamacrocyclic Lanthanide(III) Complexes: Structure, Magnetic Properties, and Temperature-Dependent Luminescence

**DOI:** 10.1021/acs.inorgchem.4c02047

**Published:** 2024-08-09

**Authors:** Paula Gawryszewska, Katarzyna Ślepokura, Jerzy Lisowski

**Affiliations:** Department of Chemistry, University of Wrocław, 14 F. Joliot-Curie, Wrocław 50-383, Poland

## Abstract

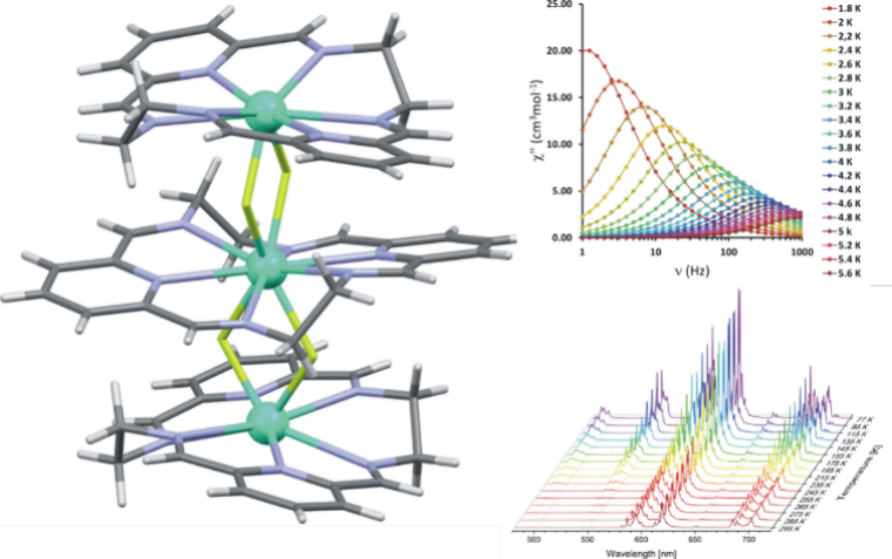

The reaction of fluoride anions with mononuclear rare-earth(III) complexes of the hexaazamacrocycle derived from 2,6-diformylpyridine and ethylenediamine affords trinuclear coordination compounds **[Ln**_**3**_**L**_**3**_**(μ**_**2**_**-F)**_**4**_**(NO**_**3**_**)**_**2**_**](NO**_**3**_**)**_**3**_. The X-ray crystal structures of these complexes show triplex cationic complexes where the three roughly parallel macrocyclic lanthanide(III) units are linked by bis-μ_2_-F bridges. The detailed analysis of the photophysical properties of the **[Eu**_**3**_**L**_**3**_**(μ**_**2**_**-F)**_**4**_**(NO**_**3**_**)**_**2**_**](NO**_**3**_**)**_**3**_**·2H**_**2**_**O** and **[Tb**_**3**_**L**_**3**_**(μ**_**2**_**-F)**_**4**_**(NO**_**3**_**)**_**2**_**](NO**_**3**_**)**_**3**_**·3H**_**2**_**O** complexes reveals different temperature dependence of luminescence intensity and luminescence decay time of the Eu(III) and Tb(III) derivatives. The spectra of mixed species of average composition **[Eu**_**1.5**_**Tb**_**1.5**_**L**_**3**_**(μ**_**2**_**-F)**_**4**_**(NO**_**3**_**)**_**2**_**](NO**_**3**_**)**_**3**_**·3H**_**2**_**O** are in accordance with the ratiometric luminescent thermometer behavior. Measurements of the direct-current (dc) magnetic susceptibility of the **[Dy**_**3**_**L**_**3**_**(μ**_**2**_**-F)**_**4**_**(NO**_**3**_**)**_**2**_**](NO**_**3**_**)**_**3**_**·2H**_**2**_**O** complex indicate possible ferromagnetic interactions between the Dy(III) ions. Alternating current (ac) susceptibility measurements of this complex indicate single-molecule magnet behavior in zero dc field with magnetic relaxation dominated by Orbach mechanism and an effective energy barrier *U*_eff_ = 12.3 cm^–1^ (17.7 K) with a pre-exponential relaxation time, τ_0_ of 7.3 × 10^–6^ s. A similar reaction of mononuclear macrocyclic complexes with a higher number of fluoride equivalents results in polymeric **{[Ln**_**3**_**L**_**3**_**(μ**_**2**_**-F)**_**5**_**](NO**_**3**_**)**_**4**_**}**_**n**_ complexes. The X-ray crystal structure of the Nd(III) derivative of this type shows trinuclear units that are additionally linked by single fluoride bridges to form a linear coordination polymer.

## Abstract 2

Macrocyclic units of lanthanide(III) hexaazamacrocyclic complex can be linked by fluoride bridges to form trinuclear complexes and 1-D coordination polymers. The mixed Tb(III)/Eu(III) trinuclear species exhibit ratiometric luminescent thermometer behavior and the trinuclear Dy(III) complex exhibits single-molecule magnet properties.

## Introduction

**Figure 1:**
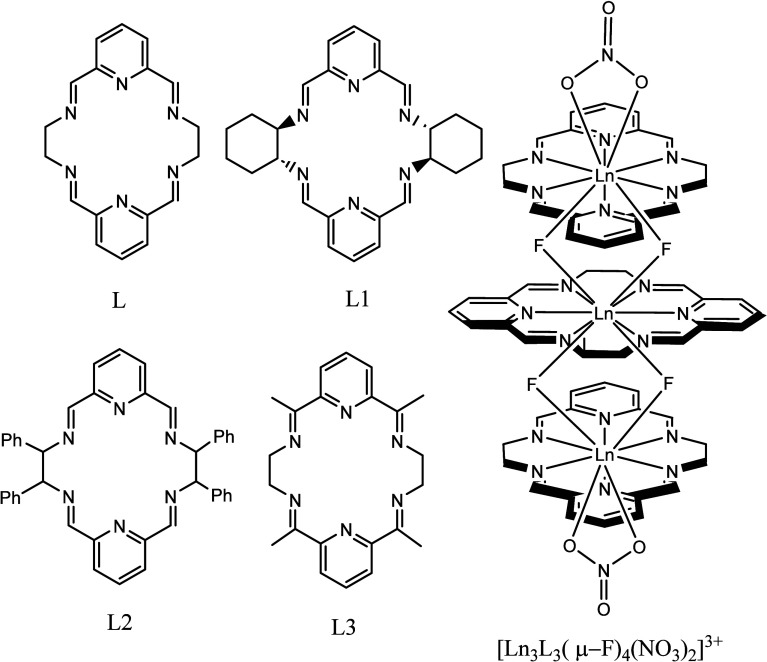
Structures of the representative hexaazamacrocyclic imines L–L3 and the cationic trinuclear lanthanide(III) complex of L.

Lanthanide(III) ions exhibit unique magnetic and spectroscopic properties that are related to applications of lanthanide(III) coordination compounds, for example, as contrast enhancing agents for magnetic resonance imaging (MRI),^1−3^ luminescent labels,^4−8^ or molecular magnets.^9−14^ These applications require tuning of the ligand environment of the Ln(III) ions and are often based on elaborate polynuclear systems. For example, polynuclear Ln(III) complexes exhibit remarkable upconversion and energy transfer effects,^15−18^ heteronuclear Ln(III) complexes can be used as ratiometric luminescent labels^19^ or molecular qubits,^20,21^ bridged dinuclear Dy(III) and Tb(III) complexes exhibit outstanding single-molecule magnet (SMM) properties,^22,23^ while contrast agents containing multiple Gd(III) ions exhibit better relaxivity due to longer correlation times of larger molecules.^2,3,24^ Due to the labile nature of Ln(III) ions, predominantly ionic character of metal–ligand bond, high coordination numbers, and lack of directional preferences for bond formation, it is difficult to control the coordination sphere of these ions, and Ln(III) complexes are often unstable. It is even more difficult to precisely control the structure of polynuclear Ln(III) coordination compounds,^20,25−27^ and for this reason, supramolecular systems of this kind are often a matter of serendipitous discovery. One way to define the coordination sphere of Ln(III) ions and form very stable complexes is to use macrocyclic ligands, as it is the case of many Gd(III)-based contrast enhancing agents.^1−3^ In particular, hexaazamacrocyclic imines derived from diamines and 2,6-diacetyl- or 2,6-diformylpyridine (Figure 1) form stable lanthanide(III) complexes due to matching the size of these relatively large macrocycles to the size of Ln(III) ions.^28−52^ Mononuclear Ln(III) complexes of this type exhibit interesting properties such as single-molecule magnet (SMM) properties,^28−34^ thermally dependent luminescence,^35^ or circularly polarized luminescence.^36,37,49^ They have also been studied as complexes that selectively interact with DNA,^47,48^ contrast agents for MRI,^49^ and molecular dopants for n-type graphene.^45^ While most of these hexaazamacrocyclic Ln(III) complexes are mononuclear, these macrocyclic units can be linked via additional bridging ligands to form dinuclear Ln(III) complexes,^37−43^ which have been studied in the context of chiral recognition, magnetism, and luminescent properties.

Fluoride anion as a hard Pearson base binds strongly to hard Ln(III) ions. However, the lanthanide(III) complexes containing organic ligands together with fluoride ligands^17,33,37,53−65^ are not numerous due to the tendency to precipitate insoluble Ln(III) fluoride salts in the synthesis of such complexes. This precipitation is prevented when the Ln(III) ion is strongly complexed by a polychelate or macrocyclic ligand. For instance, tetraaza cyclen-based macrocycles form mononuclear Ln(III) complexes with additional terminal fluoride anions^60−62^ or dinuclear complexes with a single linear μ_2_-fluorido bridge.^17,63−65^

Here, we show that under suitable conditions, linking the Ln(III) imine macrocycles by additional bridging ligands can be extended to trinuclear and polymeric systems. Thus, three mononuclear macrocyclic units containing rare-earth ions Ln(III) (Ln = Nd, Eu, Tb, and Dy) or Y(III) bound by imine hexaazamacrocycle L can be linked together by bridging fluoride anions to form a triplex complex molecule, where three relatively flat macrocyclic units are stacked on the top of each other. While dinuclear molecular Ln(III) complexes with the di-μ_2_-fluorido bridging motif are known,^37,43,55,59,66,67^ to the best of our knowledge, we present the first example of trinuclear lanthanide(III) complexes with two such bridging motifs.

## Results and Discussion

### Synthesis and X-ray Crystal Structures

While the reaction of the starting mononuclear complexes of L with 1 equiv of fluoride anions in the form of tetraethylammonium fluoride results in the formation of dinuclear complex,^37^ the application of larger amount of fluoride anions leads to the formation of trinuclear derivatives [Ln_3_L_3_(μ_2_-F)_4_(NO_3_)_2_](NO_3_)_3_·*n*H_2_O. These compounds crystallize from solution when more than a stoichiometric amount of fluoride anions is used, typically 1.8 equiv. This is in contrast to the reactions of mononuclear complexes of more substituted macrocycles such as L1 and L2, where the application of additional fluoride anions results in the coordination of fluoride at the outer axial positions of the dinuclear macrocyclic complex^37,43^ or formation of a mononuclear complex.^33^ The two macrocyclic units L1 or L2 in a double-decker dinuclear complex are rather tightly bound, which causes some additional bending of the macrocyclic ligands. Presumably, in these cases, the steric interactions of somewhat bulkier macrocycles do not allow formation of triple-decker complexes.

**Figure 2:**
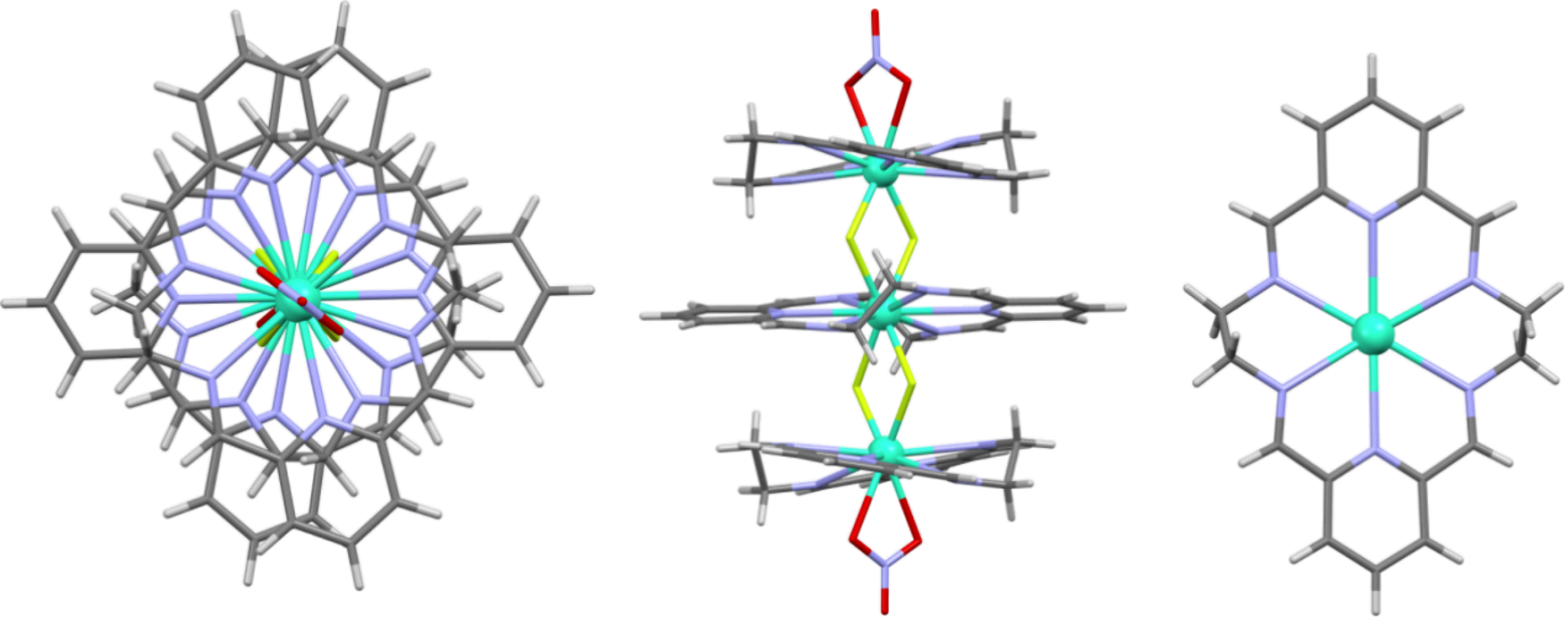
Top and side views of the trinuclear Tb(III) complex cation, together with a mononuclear building block of this complex. Color code: Tb, green; F, lime; O, red; N, blue; C, gray; H, light gray.

The crystals of [Tb_3_L_3_(μ_2_-F)_4_(NO_3_)_2_](NO_3_)_3_·5.2CHCl_3_·0.8CH_3_OH·H_2_O were obtained from a mixed chloroform/methanol solution. This trinuclear complex can be considered a triplex of lanthanide macrocyclic units (Figure 2). For the isomorphic trinuclear Nd(III), Eu(III), and Dy(III) complexes, the quality of models was lower. For this reason, only the structure of the trinuclear Tb(III) complex will be discussed here, although the crude structural models of the Nd(III), Eu(III), and Dy(III) complexes (Supporting Figures S1–S3) clearly indicate that the corresponding trinuclear complex cations are isostructural to the trinuclear Tb(III) complex cation.

All Tb(III) ions in the [Tb_3_L_3_(μ_2_-F)_4_(NO_3_)_2_]^3+^· complex ion are 10-coordinate. The coordination sphere of the central Tb(III) ion consists of six nitrogen atoms of the organic ligand and four bridging fluoride anions evenly distributed on both sides of the macrocycle. The axial ligation of the two terminal Tb(III) ions is less uniform and corresponds to two bridging fluorides on one side of the macrocycle and two oxygen atoms of the bidentate nitrate anion on the other side. As revealed by the continuous shape measurements (CShMs), calculated with the SHAPE program,^68^ the coordination polyhedra of the two crystallographically different Tb(III) cations are distorted from the ideal shapes. They may be considered as deformed tetradecahedron (*C*_2*v*_ symmetry), staggered dodecahedron (*D*_2_ symmetry) or sphenocorona (*C*_2*v*_), or more deformed bicapped square antiprism (*D*_4*d*_ symmetry). CShMs are listed in Supporting Table S2. The two planes defined by the two Tb_2_(μ_2_-F)_2_ fragments are almost perpendicular; in addition, each of these planes is roughly perpendicular to the plane defined by the adjacent bidentate nitrate anion. Thus, the coordinating atoms of axial ligands are arranged in an alternate fashion along the axis of this complex cation. Similarly, the macrocyclic ligands are also arranged in an alternating manner, with the pyridine rings of the outer macrocycles L positioned above and below the ethylene fragments of the central macrocycle L. Thus, the central macrocyclic unit is rotated almost at right angles relative to the position of the outer macrocyclic units. This rotation leads to the positioning of aliphatic C–H fragments of one macrocyclic unit close to pyridine fragment of another macrocyclic unit (H···ring plane distances are equal to ca. 2.6–2.7 Å and the H···ring centroid distances are equal to ca. 2.7–2.8 Å), which may lead to additional stabilization of the trimer via C–H···π interactions.

It should be mentioned that although macrocycle L derived from ethylenediamine is achiral, it adopts a chiral conformation when coordinated to rare-earth ions. In these complexes, the macrocycle is not flat but adopts a rigidified helical conformation in order to closely match the size of the metal ions. In this way, the monomeric complexes of macrocycle L exist as a racemic mixture of conformers with opposite helicity, which can be denoted as Λ and Δ (Supporting Figure S4). Interestingly the formation of trinuclear [Ln_3_L_3_(μ_2_-F)_4_(NO_3_)_2_]^3+^ complex cations is accompanied by the social self-sorting of such helical units. The crystal structures indicate that in the centrosymmetric crystal, the triplex complex ions are of either ΛΛΛ or ΔΔΔ conformation of the macrocyclic ligand (Supporting Figures S4 and S5).

In general, the Ln(III) complexes of hexaazamacrocyles exhibit various degrees of twisting and folding of the macrocycle. For the starting mononuclear complexes such as [DyL(NO_3_)_2_](NO_3_), with symmetric axial ligation on the two sides of the macrocycle, the fold of the macrocyclic unit is small, as shown by its X-ray crystal structure (Supporting Figure S6). Similarly, the central macrocyclic unit in the Tb(III) triplex complex is not folded, as indicated by the linear arrangement of the pyridine −Tb(III) bonds at 180° N–Tb–N angle. On the other hand, the N–Tb–N angle is equal to about 171° for the outer macrocyclic units in the triplex. This slight fold is the result of asymmetric axial ligation and steric interaction between the outer and central macrocyclic unit.

**Figure 3:**
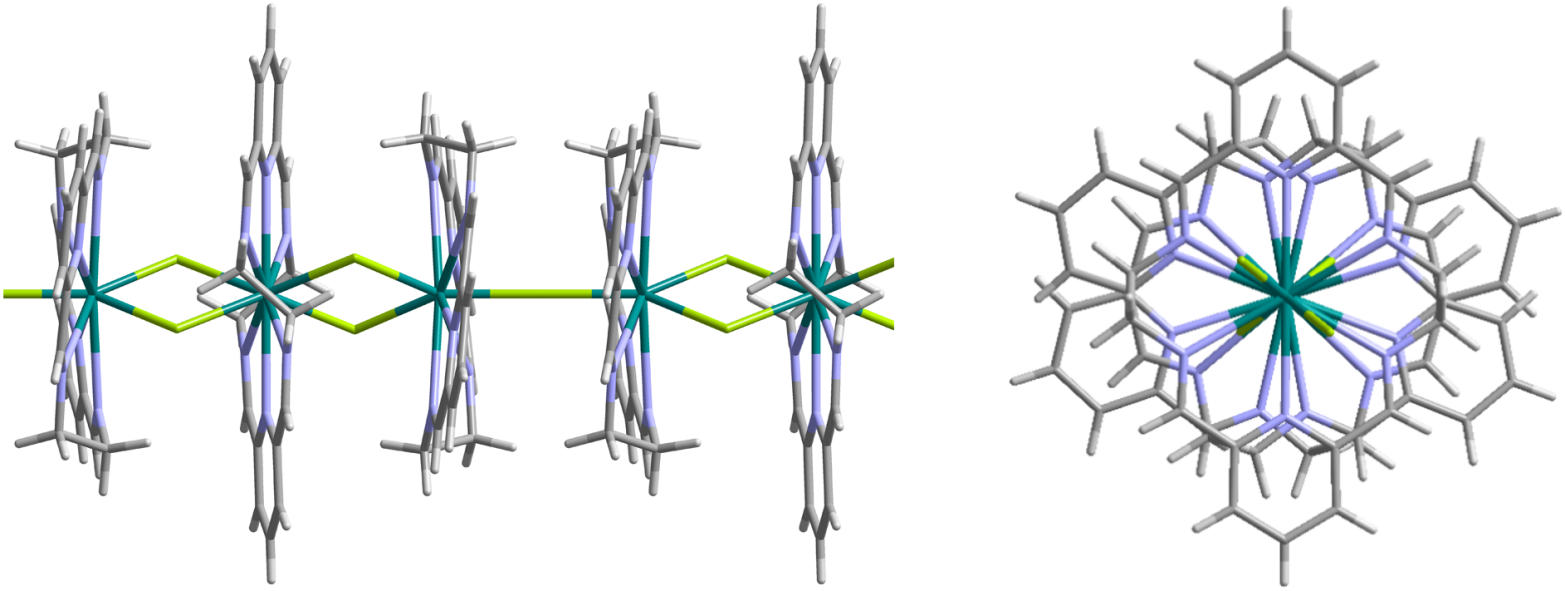
Side and top view of the fragment of the polymeric chain complex {[Nd_3_L_3_(μ_2_-F)_4_(μ-F)]^4+^}_*n*_. Color code: Nd, teal; F, lime; N, blue; C, gray; H, light gray.

The application even larger amounts of fluoride anions (2–2.2 equiv of tetraethylammonium fluoride) in the reactions with the starting mononuclear complexes enabled the isolation of single crystals of the polymeric complex {[Nd_3_L_3_(μ_2_-F)_5_](NO_3_)_4_·2.5CH_3_OH·3H_2_O}_*n*_ after prolonged standing in a mixed methanol/chloroform solution. In the case of the isomorphic Eu(III) complex, the quality of the data allowed us to solve only the crude structural model (Supporting Figure S7). The Nd(III) polymeric complex consists of trinuclear [Nd_3_L_3_(μ_2_-F)_4_]^5+^ building blocks (Supporting Figure S8) similar to the complex cations [Ln_3_L_3_(μ_2_-F)_4_(NO_3_)_2_]^3+^ of the trinuclear complexes described above. While in the trinuclear [Ln_3_L_3_(μ_2_-F)_4_(NO_3_)_2_]^3+^ cationic complex the outer Ln(III) ions are coordinated with the outer axial nitrate anions, in the polymeric cationic complex the outer Ln(III) ions of the trinuclear building blocks are coordinated with an additional bridging fluoride anion, which leads to the formation of infinite chains (Figure 3). The macrocyclic units in the [Nd_3_L_3_(μ_2_-F)_4_]^5+^ polymer fragments are mutually rotated by about 40°, much less compared to the [Ln_3_L_3_(μ_2_-F)_4_(NO_3_)_2_]^3+^ cationic complex. Remarkably, chiral self-recognition of macrocyclic units is also observed for these polymeric complex cations. Thus, all macrocyclic units within each {[Nd_3_L_3_(μ_2_-F)_4_(μ-F)]^4+^}_*n*_ chain are of the same helicity and the racemic crystal is composed of polymeric ions, where the macrocycles within one chain are of either all-Λ or all-Δ conformation (Supporting Figure S9).

The bis-μ_2_-F link between two macrocyclic units in the trinuclear fragment corresponds to a bent conformation of the bridging fluoride anions with a Nd–F–Nd angle equal to 113°. In contrast, the mono-μ-F fluoride bridge linking the trinuclear [Nd_3_L_3_(μ_2_-F)_4_]^5+^ fragments corresponds to the perfectly linear Nd–F–Nd geometry, because these Nd and F atoms are positioned on the polymeric chain axis, which is a two-fold axis. For the pairs of Nd(III) ions that are linked by two fluoride bridges, the Nd–Nd distance is equal to 3.89 Å. On the other hand, the Nd–Nd distance corresponding to a single linear fluoride bridge between trinuclear units is considerably longer and is equal to 4.57 Å. It is somewhat unexpected that within this kind of polymeric macrocyclic complex, not all pairs of macrocyclic units are linked in the same way by bis-μ_2_-F bridges. Possibly, the slight folding of the outer macrocyclic units within the repeating triplex motif makes such a link less favorable due to steric interactions. Instead, a longer link corresponding to a single linear fluoride bridge is formed, in order to ease steric interactions between the parallel macrocycles.

The influence of steric interactions on the formation of polymeric complex is also evidenced by the existence of a dinuclear cationic complex^43^ [Ln_2_(L2)_2_(μ_2_-F)_2_F_2_]^2+^ of a related macrocycle L2 derived from 1,2-diphenylethylenediamine. In this complex, two macrocyclic units are linked by a bis-μ_2_-F bridging motif, and additional fluoride anions act as outer terminal axial ligands. In principle, these terminal fluorides can become bridging ligands, and therefore, this dinuclear complex should polymerize to form a linear complex {[Ln(L2)(μ_2_-F)_2_]^2+^}_*n*_ with all macrocyclic units linked in the same bis-μ_2_-F fashion. However, in this case, the close contact between macrocyclic units within a dinuclear fragment causes them to bend outward, and this bending in turn creates a steric hindrance that prevents further aggregation.

### Magnetic Properties of the Trinuclear Dy(III) Complex

**Figure 4:**
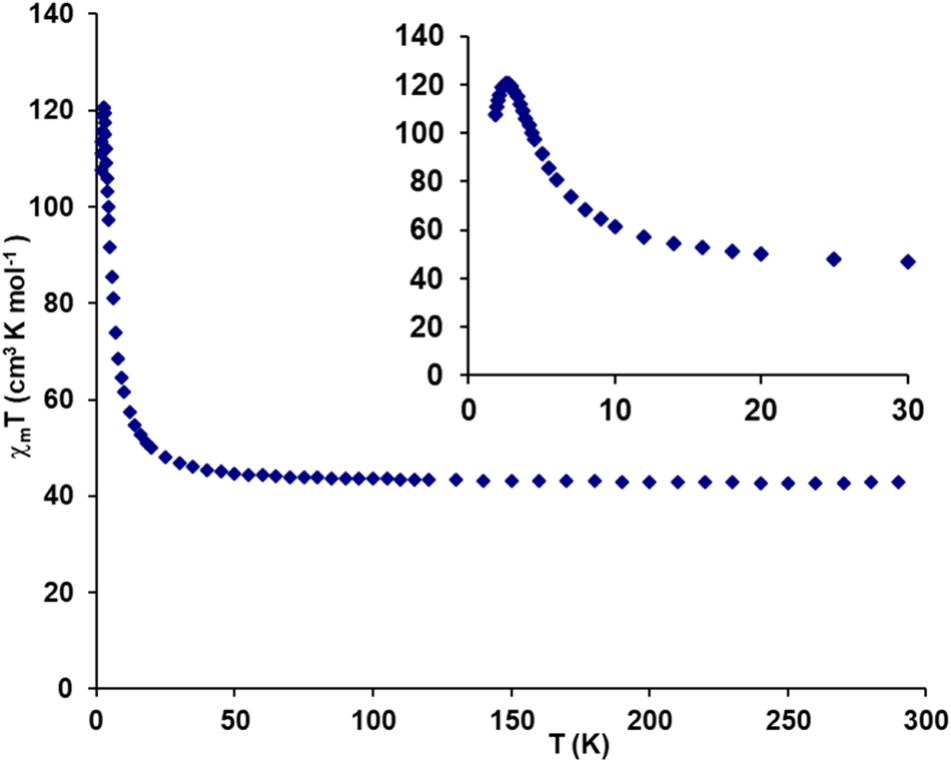
Plot of experimental χ_M_*T* vs *T* for the trinuclear **[Dy**_**3**_**L**_**3**_**(μ**_**2**_**-F)**_**4**_**(NO**_**3**_**)**_**2**_**](NO**_**3**_**)**_**3**_**·2H**_**2**_**O** complex.

Variable temperature magnetic susceptibility data for the **[Dy**_**3**_**L**_**3**_**(μ**_**2**_**-F)**_**4**_**(NO**_**3**_**)**_**2**_**](NO**_**3**_**)**_**3**_**·2H**_**2**_**O** complex were obtained in the temperature range 1.8–300 K at magnetic field 0.1 T (Figure 4). The room temperature value of the χ_M_*T* product of molar magnetic susceptibility and temperature is equal to 42.93 cm^3^ mol^–1^ K, which is close to the value of 42.51 cm^3^ mol^–1^ K expected for the three noninteracting ^6^H_15/2_ Dy(III) ions with *g* = 4/3 and *J* = 15/2. The value of this product changes little on cooling to about 50 K. Below this temperature, χ_M_*T* starts to increase and this increase accelerates below about 20 K to reach the maximum value of 120.55 cm^3^ mol^–1^ K at 2.7 K. After reaching maximum the χ_M_*T* values fall sharply on further cooling to a χ*T* value of 107.73 cm^3^ mol^–1^ K at 1.79 K. The initial increase of χ_M_*T* values suggests ferromagnetic interactions among the Dy(III) ions, while the subsequent decrease of χ_M_*T* is most likely the result of the depopulation of the Stark sublevels of the ^6^H_15/2_ state. Similar ferromagnetic interactions were suggested for the dinuclear Dy(III) complex with a similar hexaazamacrocycle and analogous bis-μ_2_-F bridging motif^43^ and related dinuclear complexes with bis-μ_2_–OH bridging motif.^41^

Magnetization (M) data were collected over the 0–70 kOe field range at 2, 3, and 5 K. The magnetization increases rapidly at low field to reach a value of 19.63 μ_B_ at 70 kOe, which is lower than the expected saturation value, most likely due to the crystal-field effect and/or low-lying excited states. The isotherms of magnetization M versus field H are not superimposable (Supporting Figure S10) suggesting the presence of a significant magnetic anisotropy and/or low-lying excited states.

**Figure 5:**
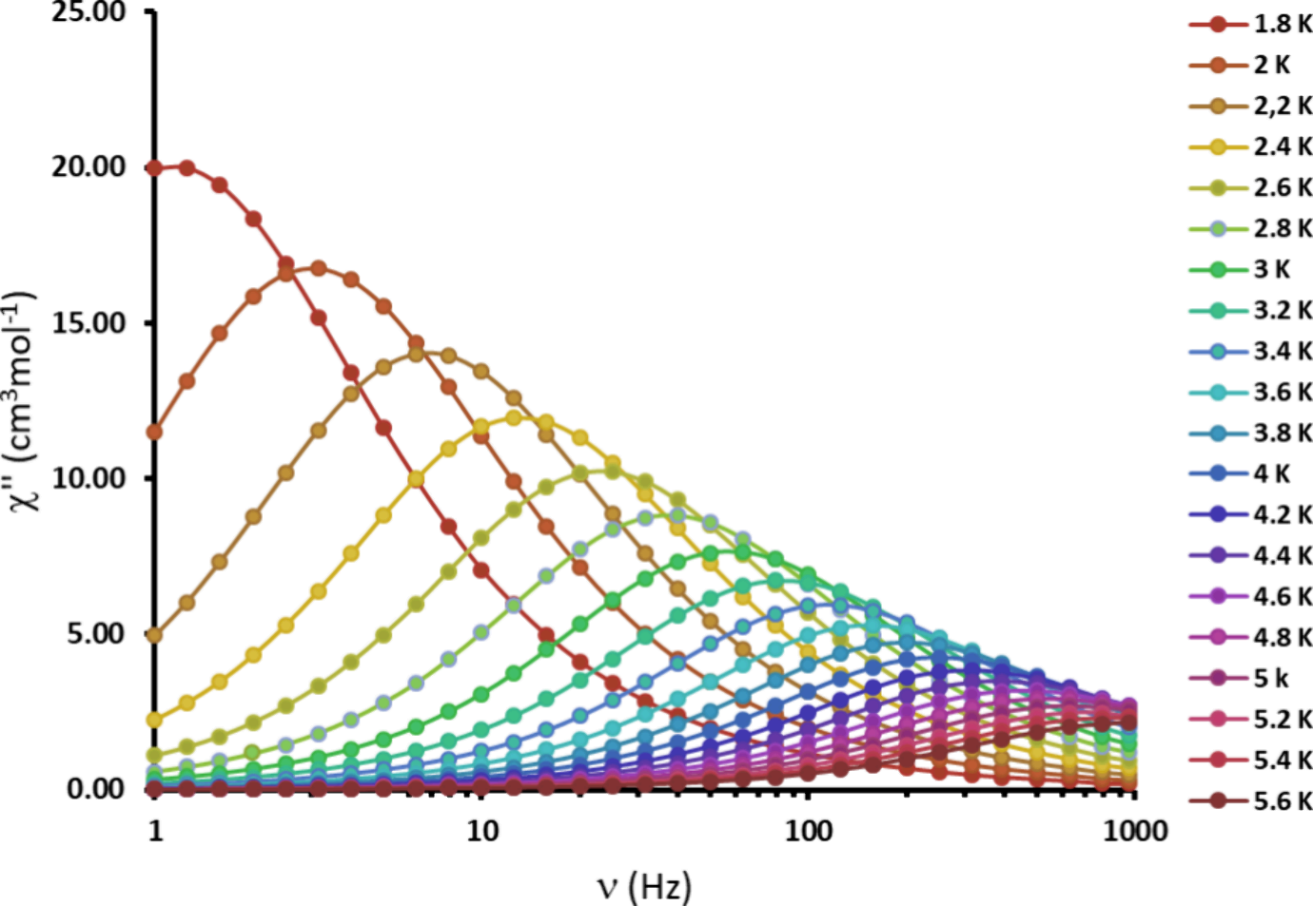
Frequency dependence of the imaginary component of ac susceptibility (χ″) for the trinuclear **[Dy**_**3**_**L**_**3**_**(μ**_**2**_**-F)**_**4**_**(NO**_**3**_**)**_**2**_**](NO**_**3**_**)**_**3**_**·2H**_**2**_**O** complex under zero dc field at ac frequencies of 1–960 Hz in the temperature range of 1.8–5.6 K.

The trinuclear Dy(III) complex **[Dy**_**3**_**L**_**3**_**(μ**_**2**_**-F)**_**4**_**(NO**_**3**_**)**_**2**_**](NO**_**3**_**)**_**3**_**·2H**_**2**_**O** exhibits clear single molecule magnet properties, as indicated by ac susceptibility measurements as a function of temperature at different frequencies under a zero dc field. This compound exhibits frequency and temperature dependent in-phase, χ′, and out-of-phase, χ″ ac susceptibility (Figure 5 and Supporting Figures S11–S13). Clear maxima for the out-of-phase ac susceptibility signals indicate slow relaxation of the magnetization (Figure 5) and SMM properties at a zero dc field.

The distribution of the relaxation time was determined on the basis of the Cole–Cole plot (Supporting Figure S14) using CC-Fit2 software.^69^ The data were fitted using a generalized one process Debye model and gave α Cole–Cole parameter values ranging from 0.05 to 0.15 at 1.8–5.4 K, corresponding to a narrow relaxation time distribution.

**Figure 6:**
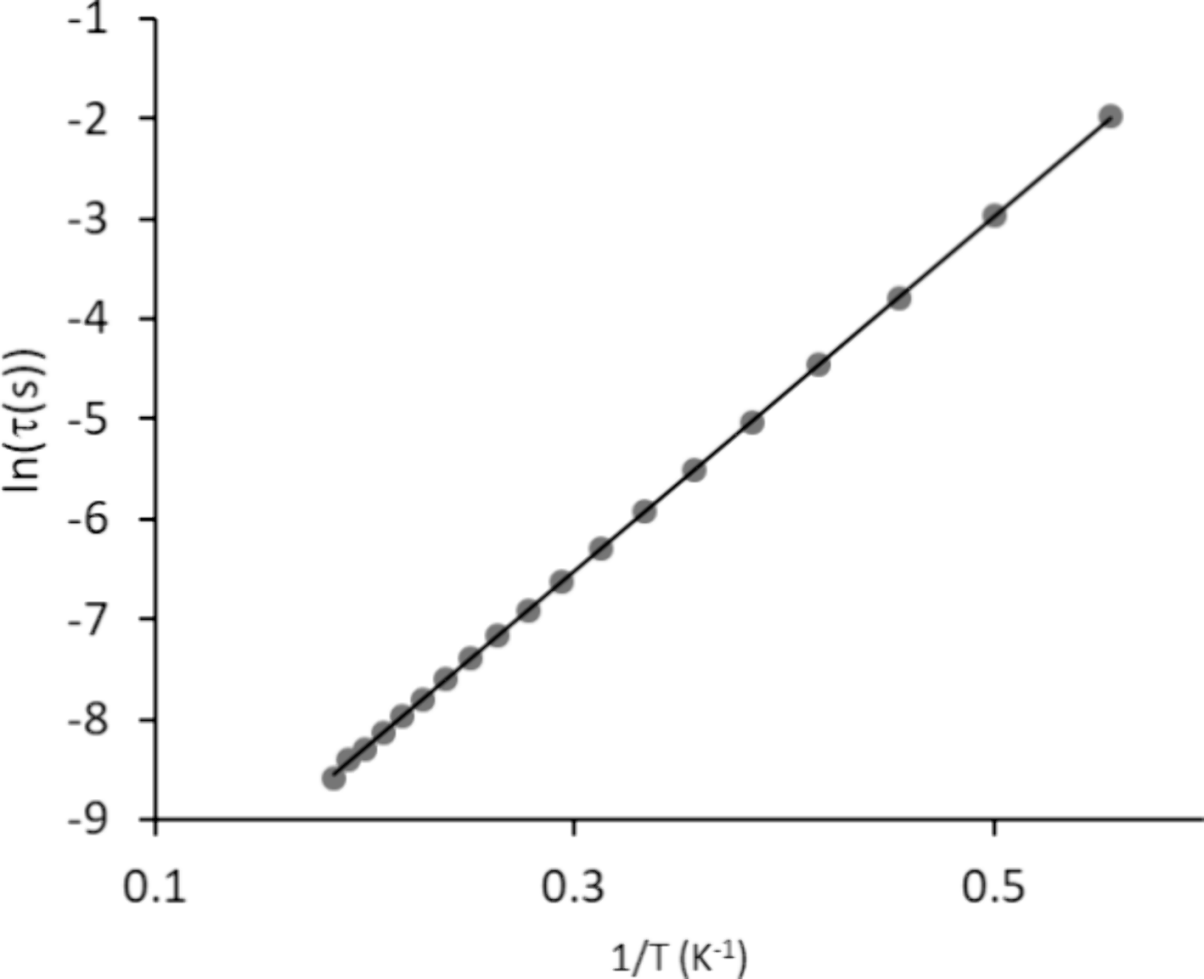
Temperature dependence of the magnetization relaxation time for the trinuclear **[Dy**_**3**_**L**_**3**_**(μ**_**2**_**-F)**_**4**_**(NO**_**3**_**)**_**2**_**](NO**_**3**_**)**_**3**_**·2H**_**2**_**O** complex as ln(τ) versus *T*^–1^ plot.

Temperature-dependent magnetic relaxation is in general dependent on multiple relaxation mechanisms, as given by the equation τ^–1^ = τ_0_^–1^ exp(−*U*_eff_/*K*_B_*T*) + *CT^n^*+ τ_QTM_^–1^, where the three terms correspond to Orbach mechanism, Raman mechanism, and quantum tunneling of magnetization, respectively.^11,14,70,71^ The plot of natural logarithm of the relaxation time ln(τ) vs inverse temperature was used in order to analyze the relaxation mechanisms. For the discussed trinuclear complex, the linear Arrhenius plot (Figure 6) is consistent with the first term only, and indicates that the relaxation is governed by the Orbach process. Arrhenius law is well obeyed in the range of studied temperature and gives the energy barrier *U*_eff_ = 12.3 cm^–1^ (17.7 K) with a pre-exponential relaxation time, τ_0_ of 7.3 × 10^–6^ s. The moderate value of *U*_eff_ is related to the deviation of the symmetry of this trinuclear complex from strict axial symmetry, while the lack of typical Raman contribution may suggest a relatively rigid system.

### Luminescence Properties of Trinuclear Eu(III) and Tb(III) Complexes

Three fluoride-bridged lanthanide ions, coordinated by azamacrocyclic ligands, offer the possibility of constructing systems with diverse luminescent properties. Photophysical properties of lanthanide(III) complexes are dependent on the choice of Ln(III) ions and on competing intramolecular nonradiative energy transfer processes. In the **[Ln**_**3**_**L**_**3**_**(μ**_**2**_**-F)**_**4**_**(NO**_**3**_**)**_**2**_**](NO**_**3**_**)**_**3**_**·3H**_**2**_**O** trinuclear coordination compounds, nonradiative interion Ln–Ln energy transfer (IIET), forward ligand-to-metal energy transfer (FET), and backward energy transfer from the emissive (or other excited) Ln(III) level to the excited ligand state (BET) may occur. Moreover, by designing an appropriate selection of two different Ln(III) ions, one of which is characterized by strongly temperature-dependent luminescence, compounds with potential applications in luminescence thermometry can be obtained.^72^ Taking into account the intensity of luminescence and the aspect of optical thermometry, an analysis of the photophysical properties of the **[Eu**_**3**_**L**_**3**_**(μ**_**2**_**-F)**_**4**_**(NO**_**3**_**)**_**2**_**](NO**_**3**_**)**_**3**_**·2H**_**2**_**O**, **[Tb**_**3**_**L**_**3**_**(μ**_**2**_**-F)**_**4**_**(NO**_**3**_**)**_**2**_**](NO**_**3**_**)**_**3**_**·3H**_**2**_**O** and **[Eu**_**1.5**_**Tb**_**1.5**_**L**_**3**_**(μ**_**2**_**-F)**_**4**_**(NO**_**3**_**)**_**2**_**](NO**_**3**_**)**_**3**_**·3H**_**2**_**O** compounds was carried out. Eu(III) and Tb(III) have the largest energy gap among Ln(III) (excluding Gd(III)) between the emitting level and the nearest level with a lower energy, which makes them characterized by the most intense emission. This emission can be additionally sensitized by organic ligands, which enhance its brightness considerably.

**Figure 7:**
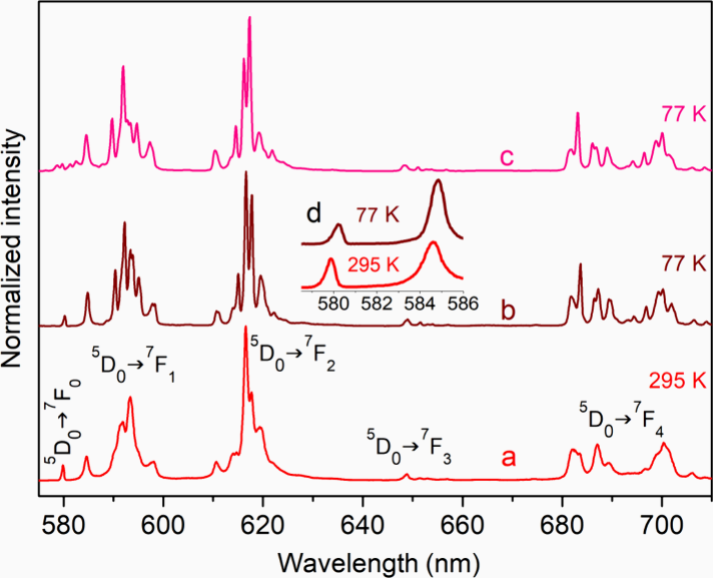
Emission spectra of (a) **[Eu**_**3**_**L**_**3**_**(μ**_**2**_**-F)**_**4**_**(NO**_**3**_**)**_**2**_**](NO**_**3**_**)**_**3**_**·2H**_**2**_**O** at 295 K (λ_exc_ = 337 nm); (b) **[Eu**_**3**_**L**_**3**_**(μ**_**2**_**-F)**_**4**_**(NO**_**3**_**)**_**2**_**](NO**_**3**_**)**_**3**_**·3H**_**2**_**O** at 77 K (λ_exc_ = 330 nm); (c) [**Eu**_**1.5**_**Tb**_**1.5**_**L**_**3**_**(μ**_**2**_**-F)**_**4**_**(NO**_**3**_**)**_**2**_**](NO**_**3**_**)**_**3**_**·3H**_**2**_**O** at 77 K (λ_exc_ = 337 nm). (d) Inset shows ^5^D_0_ → ^7^F_0_ transition for **[Eu**_**3**_**L**_**3**_**(μ**_**2**_**-F)**_**4**_**(NO**_**3**_**)**_**2**_**](NO**_**3**_**)**_**3**_**·3H**_**2**_**O**.

Figure 7 and Supporting Figure S15 show emission spectra of **[Eu**_**3**_**L**_**3**_**(μ**_**2**_**-F)**_**4**_**(NO**_**3**_**)**_**2**_**](NO**_**3**_**)**_**3**_**·2H**_**2**_**O**, **[Eu**_**1.5**_**Tb**_**1.5**_**L**_**3**_**(μ**_**2**_**-F)**_**4**_**(NO**_**3**_**)**_**2**_**](NO**_**3**_**)**_**3**_**·3H**_**2**_**O**, and **[Tb**_**3**_**L**_**3**_**(μ**_**2**_**-F)**_**4**_**(NO**_**3**_**)**_**2**_**](NO**_**3**_**)**_**3**_**·3H**_**2**_**O** at 295 and 77 K. These spectra exhibit typical ^5^D_0_ Eu(III) and ^5^D_4_ Tb(III) (Supporting Figure S15) emissions with dominant transitions ^5^D_0_ → ^7^F_2_ and ^5^D_4_ → ^7^F_5_, respectively.

For **[Eu**_**3**_**L**_**3**_**(μ**_**2**_**-F)**_**4**_**(NO**_**3**_**)**_**2**_**](NO**_**3**_**)**_**3**_**·2H**_**2**_**O**, two bands are observed corresponding to the ^5^D_0_ → ^7^F_0_ transition with maxima of 17,245 and 17,105 cm^–1^ at 295 K (see the inset in Figure 7). The spectral range of the latter band makes it difficult to assign it to a given transition. It can be considered alternatively as a component of the ^5^D_0_ → ^7^F_1_ transition. For instance, such an assignment of the 17,164 cm^–1^ band was proposed by Binnemans^73^ for the Eu(III) compound with the Schiff base ligand, based on considerations discussed in ref^74^ and calculations of crystal field parameters. However, in our case, two different Eu(III) sites are present in the crystal structure with a different chemical environment and low local symmetry with distorted coordination polyhedra, as discussed above on the basis of continuous shape measurements (Supporting Table S2). This argument, as well as the doubling of the expected number of Stark components of the ^5^D_0_ → ^7^F_1_ band in the 16,714–16,988 cm^–1^ range, in addition to the bathochromic shift of peaks with maxima at 17,245 and 17,105 cm^–1^, allowed us to assign the latter band to the ^5^D_0_ → ^7^F_0_ transition. The ^5^D_0_ → ^7^F_0_ transition is forbidden by the Judd–Ofelt forced dipole mechanism and occurs as a result of J-mixing of ^7^F_0_ with ^7^F_2_ or of mixing of low-lying charge-transfer states into the wave functions of the 4f^6^ configuration. This transition is present in emission and excitation spectra of Eu(III) in the *C*_*nv*_, *C*_6_, *C*_3_, *C*_4_, *C*_s_, and *C*_1_ point groups.^75,76^ Despite numerous studies, including the correlation of the frequency of the ^5^D_0_ → ^7^F_0_ Eu^3+^ transition with the sum of derived nephelauxetic parameters of the ligating atoms in Eu^3+^ complexes,^77^ there seems to be no simple relationship between the type of ligand and the spectral position of the ^5^D_0_ → ^7^F_0_ transition, which depends on many factors. It has been shown that the energy of this band with respect to the free ion is the resultant of interelectronic repulsion and spin–orbit coupling.^75,76^ For these reasons, it is difficult to determine which of the two Eu(III) sites corresponds to the lower-energy peak ^5^D_0_ → ^7^F_0_. The half-widths of the two ^5^D_0_ → ^7^F_0_ bands are 15 (maximum at 17245 cm^–1^) and 31 cm^–1^ (maximum at 17105 cm^–1^) at room temperature. These values are quite large and may be due to the disorder present in the crystal structure.^78^ As the temperature decreases, a decrease in the half-width only for the second band from 31 to 22 cm^–1^ is observed as well as a shift of both bands toward lower energies. Such a shift is a frequently observed phenomenon^79,80^ and is due to interactions between f electrons, which occur due to thermal expansion of the compound and spin–orbit coupling.

The ^5^D_0_ emission decay time of **[Eu**_**3**_**L**_**3**_**(μ**_**2**_**-F)**_**4**_**(NO**_**3**_**)**_**2**_**](NO**_**3**_**)**_**3**_**·2H**_**2**_**O** monitored within the ^5^D_0_ → ^7^F_4_ and ^5^D_0_ → ^7^F_2_ transitions is independent of temperature and excitation wavelength (direct excitation λ_exc_ = 465 nm, indirect excitation λ_exc_ = 330 nm) (see Table 1 and Supporting Figure S16). The lack of temperature dependence of the decay time indicates the absence of thermally activated nonradiative processes including the emitting ^5^D_0_ level. Despite the two Eu(III) sites, the decay profiles of all recorded decay curves are monoexponential, as can be seen in Supporting Figure S16. The monoexponential decay profile of the ^5^D_0_ emission may be due to the similar contribution of nonradiative processes to the depopulation of Eu1 and Eu2 emitting states and/or fast IIET. It should be remembered that due to the small Ln-Ln distance of 3.8 Å, intramolecular IIET is present. Therefore, despite the presence of two Eu(III) sites, the emission spectrum of the compound at 295 K was used to calculate the spontaneous emission coefficients (A_0λ_) and the radiative lifetime, where the magnetic dipole transition ^5^D_0_ → ^7^F_1_ was used as a reference.^81^ The data obtained and the experimental emission decay time allowed us to calculate the intrinsic emission quantum yield () and the sensitization efficiency of the ^5^D_0_ emission (η). The value of 1.5 was assumed as the refractive index, which is commonly used for coordination compounds in the solid state.^5^ The photophysical data are summarized in Table 2. The  and η values were calculated as 38 and 21%, respectively. The **[Eu**_**3**_**L**_**3**_**(μ**_**2**_**-F)**_**4**_**(NO**_**3**_**)**_**2**_**](NO**_**3**_**)**_**3**_**·3H**_**2**_**O** compound is characterized by a medium emission sensitization efficiency, which is reflected in the value of the overall emission quantum yield () of 8%. It seems that at a small ligand–Eu distance, additional nonradiative processes related to depopulation of ligand states (singlet S_1_ or triplet T_1_) by ligand–metal charge transfer (LMCT) state or effective BET from Eu(III) excited levels other than ^5^D_0_ to S_1_ and/or T_1_ states must be responsible for such  value. In the case of various Eu(III) compounds, the contribution of LMCT in reducing the sensitization efficiency is a common phenomenon.^81−84^

**Table 1:** Decay Time of ^5^D_0_ and ^5^D_4_ Emission for **[Eu**_**3**_**L**_**3**_**(μ**_**2**_**-F)**_**4**_**(NO**_**3**_**)**_**2**_**](NO**_**3**_**)**_**3**_**·2H**_**2**_**O** and **[Tb**_**3**_**L**_**3**_**(μ**_**2**_**-F)**_**4**_**(NO**_**3**_**)**_**2**_**](NO**_**3**_**)**_**3**_**·3H**_**2**_**O** in the Solid State at 295 and 77K^a^

parameter	τ (μs)		
λ_excitation_, temperature	Eu compound ^5^D_0_→^7^F_4_	Eu compound ^5^D_0_→^7^F_2_	Tb compound ^5^D_4_→^7^F_5_
ligand excitation^b^, 295 K	1890 ± 4	1840 ± 4	19 ± 0.3 (95%)
			41 ± 13 (5%)
ligand excitation^b^, 77 K	1817 ± 4	1751 ± 4	520 ± 29 (7%)
			2366 ± 10 (93%)
465 nm (Eu), 487 nm (Tb), 295 K	1899 ± 5	1844 ± 4	nonexponential
465 nm (Eu), 487 nm (Tb), 77 K	1837 ± 6	1707 ± 4	547 ± 57 (5%)
			2383 ± 12 (95%)

a Emission decay times were monitored within the ^5^D_0_ → ^7^F_4_ (λ_em_ = 701.8 nm), ^5^D_0_ → ^7^F_2_ (λ_em_ = 616.6 nm), and ^5^D_4_ → ^7^F_5_ (λ_em_ = 545.3 nm) transitions.

b λ_exc_ corresponded to the maximum of the ligand band, which was slightly different for individual compounds and different lanthanides (applies to **[Tb**_**1.5**_**Eu**_**1.5**_**L**_**3**_**(μ**_**2**_**-F)**_**4**_**(NO**_**3**_**)**_**2**_**](NO**_**3**_**)**_**3**_)**·3H**_**2**_**O** (lambda range 330–337 nm).

**Table 2:** Photophysical Data of **[Eu**_**3**_**L**_**3**_**(μ**_**2**_**-F)**_**4**_**(NO**_**3**_**)**_**2**_**](NO**_**3**_**)**_**3**_**·2H**_**2**_**O** in the Solid State

[Eu_3_L_3_(μ_2_-F)_4_(NO_3_)_2_](NO_3_)_3_	
parameters	value
*A*_rad_ (s^–1^)	205
*A*_nrad_ (s^–1^)	330
*A* (s^–1^)	535
	8
(%)	38
η (%)	21

**Figure 8:**
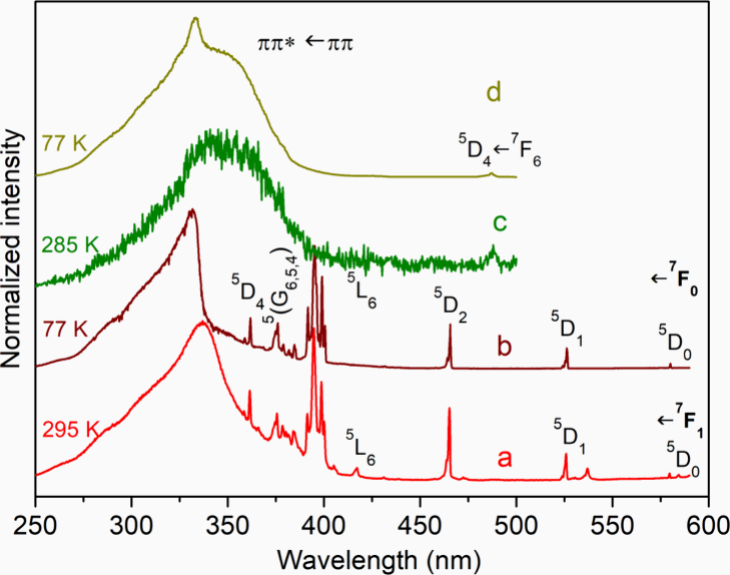
Excitation spectra of (a) **[Eu**_**3**_**L**_**3**_**(μ**_**2**_**-F)**_**4**_**(NO**_**3**_**)**_**2**_**](NO**_**3**_**)**_**3**_**·2H**_**2**_**O** at 295 K (λ_mon_ = 616.6 nm); (b) **[Eu**_**3**_**L**_**3**_**(μ**_**2**_**-F)**_**4**_**(NO**_**3**_**)**_**2**_**](NO**_**3**_**)**_**3**_**·3H**_**2**_**O** at 77 K (λ_mon_ = 616.6 nm); (c) [**Tb**_**2**_**L**_**3**_**(μ**_**2**_**-F)**_**4**_**(NO**_**3**_**)**_**2**_**](NO**_**3**_**)**_**3**_**·3H**_**2**_**O** at 295 K (λ_mon_ = 545.3 nm); (d) [**Tb**_**2**_**L**_**3**_**(μ**_**2**_**-F)**_**4**_**(NO**_**3**_**)**_**2**_**](NO**_**3**_**)**_**3**_**·3H**_**2**_**O** at 77 K (λ_mon_ = 545.3 nm).

The ligand-to-lanthanide energy-transfer process also takes place for **[Tb**_**3**_**L**_**3**_**(μ**_**2**_**-F)**_**4**_**(NO**_**3**_**)**_**2**_**](NO**_**3**_**)**_**3**_**·3H**_**2**_**O**. This is reflected in the excitation spectra. Figure 8 shows excitation spectra for **[Eu**_**3**_**L**_**3**_**(μ**_**2**_**-F)**_**4**_**(NO**_**3**_**)**_**2**_**](NO**_**3**_**)**_**3**_**·2H**_**2**_**O** and **[Tb**_**3**_**L**_**3**_**(μ**_**2**_**-F)**_**4**_**(NO**_**3**_**)**_**2**_**](NO**_**3**_**)**_**3**_**·3H**_**2**_**O** at 295 and 77 K, with a dominant broad band in the 250–400 nm range, which corresponds to ligand absorption. The ^5^D_4_ emission intensity of **[Tb**_**3**_**L**_**3**_**(μ**_**2**_**-F)**_**4**_**(NO**_**3**_**)**_**2**_**](NO**_**3**_**)**_**3**_**·3H**_**2**_**O** is strongly temperature-dependent, as can be seen in Figure S17. There is a huge rise in the integral emission intensity, which increases by as much as 450 times from a temperature of 295 to 77 K (see Supporting Figures S17 and S18).

It has been found that in the temperature range 300–250 K, the ^5^D_4_ emission intensity is low and increases slightly. It systematically increases in the 250–100 K range, and then, a sharp rise in intensity is observed at 77 K (see Supporting Figures S17 and S19).

A strong temperature dependence is also observed for ^5^D_4_ emission decay times for **[Tb**_**3**_**L**_**3**_**(μ**_**2**_**-F)**_**4**_**(NO**_**3**_**)**_**2**_**](NO**_**3**_**)**_**3**_**·3H**_**2**_**O** (see Table 1 and Supporting Figures S20–S22). The decay profiles of decay curves recorded under both indirect and direct excitation are two-exponential, as shown in Supporting Figure S20. The length of the emission decay time strongly depends on the temperature, and the character of these relationships for the two different excitation wavelengths is similar, which for the individual components of decay time under indirect and direct excitation is illustrated in Supporting Figures S21 and S22, respectively. From about 260 K upward, clear differences are observed in the values of the decay time depending on the excitation wavelength, as can be seen in Table 1 and Figure S20, as well as from a comparison of Supporting Figures S21 and S22. The decay time with ligand excitation is longer than with direct Tb(III) excitation (λ_exc_ = 487.3 nm), which is due to the presence of the T_1_ state in near-resonance with the ^5^D_4_ level and the FET process.^85^ The key issue for the Tb(III) ion is the position of the ligand triplet state. If the T_1_ energy is small enough to establish thermal equilibrium between the T_1_ and ^5^D_4_ levels, a temperature dependence of the decay time and intensity of ^5^D_4_ emission is observed as a result of BET from the Tb(III) emitting state to the ligand T_1_ state.^86,87^ It should also be remembered that the efficiency of BET is influenced by activation energy and frequency factor in addition to the T_1_ energy.^88,89^ In the case of **[Tb**_**3**_**L**_**3**_**(μ**_**2**_**-F)**_**4**_**(NO**_**3**_**)**_**2**_**](NO**_**3**_**)**_**3**_**·3H**_**2**_**O**, both Tb1 and Tb2 sites contribute to the BET process, and given the short Tb–Tb distance, IIET between the Tb(III) ions also occurs. Unfortunately, we are unable to determine the energy of the ligand’s T_1_ state because the **[Y**_**3**_**L**_**3**_**(μ**_**2**_**-F)**_**4**_**(NO**_**3**_**)**_**2**_**](NO**_**3**_**)**_**3**_**·3H**_**2**_**O** complex does not exhibit phosphorescence. The temperature dependence of the ^5^D_4_ emission intensity and its decay time is much stronger for [**Tb**_**2**_**L**_**3**_**(μ**_**2**_**-F)**_**4**_**(NO**_**3**_**)**_**2**_**](NO**_**3**_**)**_**3**_**·3H**_**2**_**O** than for the mononuclear compounds [TbL^1^NO_3_]^+^ and [TbL^1^NCS_3_]^24^ with an analogous ligand. The reason must be the much smaller energy difference between the ligand T_1_ and ^5^D_4_ Tb(III) states for [**Tb**_**2**_**L**_**3**_**(μ**_**2**_**-F)**_**4**_**(NO**_**3**_**)**_**2**_**](NO**_**3**_**)**_**3**_**·3H**_**2**_**O** than for the mononuclear compounds, as evidenced by, among other things, the much longer decay time of ^5^D_4_ emission for [TbL^1^NO_3_]^+^ and [TbL^1^NCS_3_] at 300 K (165 and 113 μs, respectively).^35^

**Figure 9:**
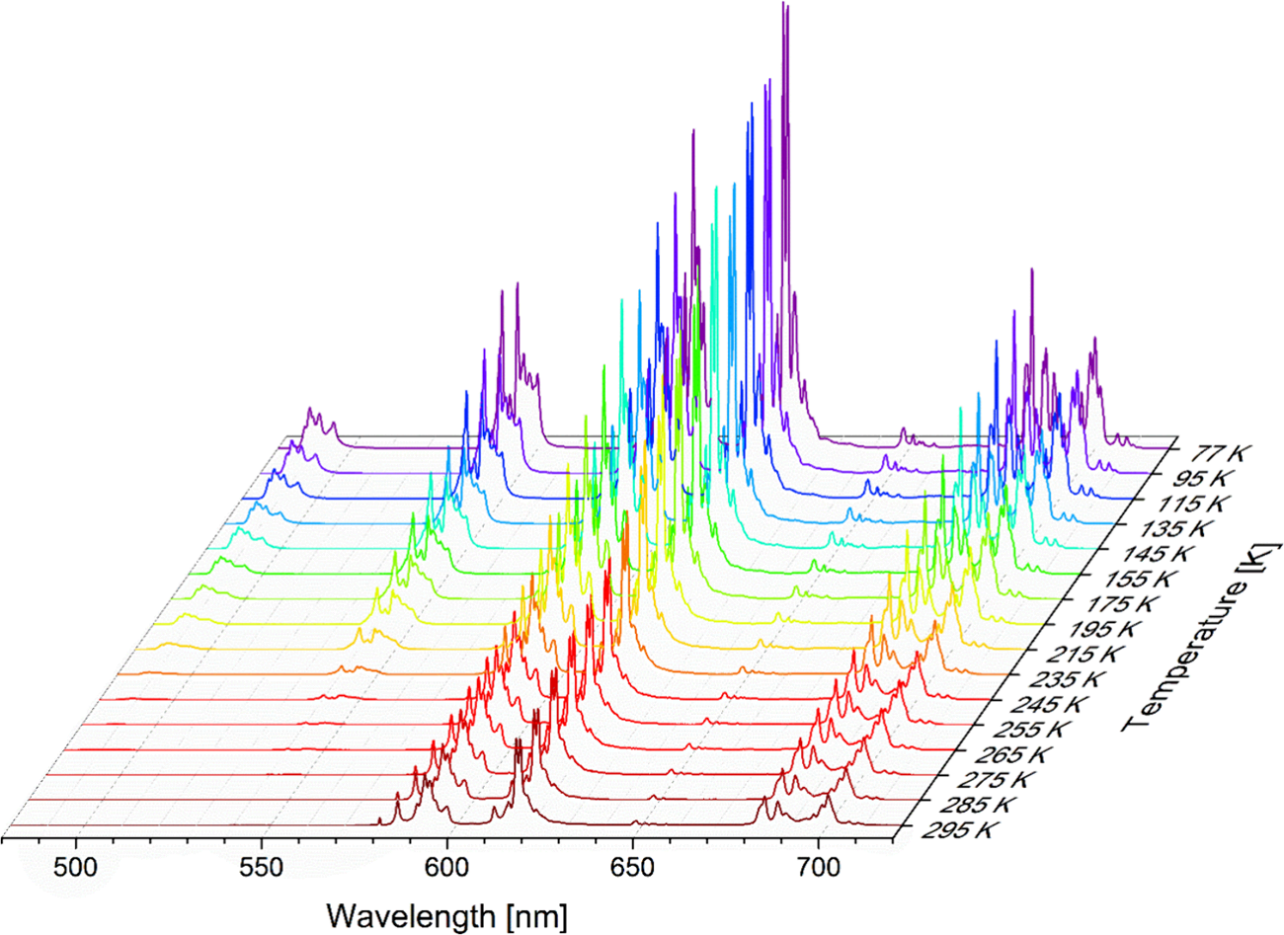
Emission spectra of **[Eu**_**1.5**_**Tb**_**1.5**_**L**_**3**_**(μ**_**2**_**-F)**_**4**_**(NO**_**3**_**)**_**2**_**](NO**_**3**_**)**_**3**_**·3H**_**2**_**O**, with λ_exc_ = 344 nm.

Due to the very strong temperature dependence of Tb(III) emission, in order to obtain a system with a thermometric response based on dual-center emission, a complex with an equimolar amount of Tb(III) and Eu(III) of an average formula of **[Eu**_**1.5**_**Tb**_**1.5**_**L**_**3**_**(μ**_**2**_**-F)**_**4**_**(NO**_**3**_**)**_**2**_**](NO**_**3**_**)**_**3**_**·3H**_**2**_**O** was synthesized. In such a system, we are dealing with the presence of **[Eu**_**2**_**Tb**_**1**_**L**_**3**_**(μ**_**2**_**-F)**_**4**_**(NO**_**3**_**)**_**2**_**](NO**_**3**_**)**_**3**_, **[Eu**_**1**_**Tb**_**2**_**L**_**3**_**(μ**_**2**_**-F)**_**4**_**(NO**_**3**_**)**_**2**_**](NO**_**3**_**)**_**3**_, and **[Tb**_**3**_**L**_**3**_**(μ**_**2**_**-F)**_**4**_**(NO**_**3**_**)**_**2**_**](NO**_**3**_**)**_**3**_, **[Eu**_**3**_**L**_**3**_**(μ**_**2**_**-F)**_**4**_**(NO**_**3**_**)**_**2**_**](NO**_**3**_**)**_**3**_ compounds resulting from statistical distribution and thus with numerous intramolecular energy transfer processes of the type: ligand → Tb(III), Tb(III) → ligand, ligand → Eu(III), Tb(III) → Tb(III), Tb(III) → Eu(III), Eu(III) → Eu(III) and perhaps Eu(III) → ligand. The Tb → Eu energy transfer is evidenced by the presence of transitions: ^5^D_4_ ← ^7^F_6_ in the **[Eu**_**1.5**_**Tb**_**1.5**_**L**_**3**_**(μ**_**2**_**-F)**_**4**_**(NO**_**3**_**)**_**2**_**](NO**_**3**_**)**_**3**_**·3H**_**2**_**O** excitation spectra when monitoring Eu(III) emission (Supporting Figure S23), ^5^D_0_ → ^7^F_J_ (J = 1,2,4) in the **[Eu**_**1.5**_**Tb**_**1.5**_**L**_**3**_**(μ**_**2**_**-F)**_**4**_**(NO**_**3**_**)**_**2**_**](NO**_**3**_**)**_**3**_**·3H**_**2**_**O** emission spectra (Supporting Figure S24) under Tb(III) excitation (λ_exc_ = 487 nm) as well as shortened ^5^D_4_ emission decay times compared to **[Tb**_**3**_**L**_**3**_**(μ**_**2**_**-F)**_**4**_**(NO**_**3**_**)**_**2**_**](NO**_**3**_**)**_**3**_**·3H**_**2**_**O** decays, (see Table 3). Emission spectrum of **[Tb**_**3**_**L**_**3**_**(μ**_**2**_**-F)**_**4**_**(NO**_**3**_**)**_**2**_**](NO**_**3**_**)**_**3**_**·3H**_**2**_**O** (λ_exc_ = 487 nm) is also included in Supporting Figure S24, for comparison. Figure 9 and Figure S25 show the emission and excitation spectra of **[Eu**_**1.5**_**Tb**_**1.5**_**L**_**3**_**(μ**_**2**_**-F)**_**4**_**(NO**_**3**_**)**_**2**_**](NO**_**3**_**)**_**3**_**·3H**_**2**_**O** as a function of temperature. In addition to the strong temperature dependence of the Tb(III) emission, a relatively weak dependence for Eu(III) emission is also observed. A comparison of the type and magnitude of changes in luminescence intensity as a function of temperature are presented in Supporting Figure S26. The integral intensity of Eu(III) emission calculated for the ^5^D_0_ → ^7^F_4_ transition increases by 2.6 times in the 295–77 K range, which is much less than the integral intensity of Tb(III) emission (an increase of 450 times). The relative ratio of ^5^D_4_ → ^7^F_5_/^5^D_0_ → ^7^F_2_ intensities for **[Eu**_**1.5**_**Tb**_**1.5**_**L**_**3**_**(μ**_**2**_**-F)**_**4**_**(NO**_**3**_**)**_**2**_**](NO**_**3**_**)**_**3**_**·3H**_**2**_**O** can be used for the absolute temperature measurement. The value of this ratio depends on the balance between rates of all the nonradiative transitions mentioned above. Some of the main processes responsible for the temperature dependence of ^5^D_4_ → ^7^F_5_/^5^D_0_ → ^7^F_4_ are BET and Tb → Eu energy transfer.

**Table 3:** Decay Time of ^5^D_0_ and ^5^D_4_ Emission for **[Eu**_**1.5**_**Tb**_**1.5**_**L**_**3**_**(μ**_**2**_**-F)**_**4**_**(NO**_**3**_**)**_**2**_**](NO**_**3**_**)**_**3**_**·3H**_**2**_**O** in the Solid State at 295 and 77K^a^

τ (μs)	
^5^D_0_ emission	^5^D_4_ emission
λ_exc_ = 487 nm	λ_exc_ = 337 nm
1948 ± 6 (295 K)	19.0 ± 0.3 (93%) (295 K)
1936 ± 6 (77 K)	37.0 ± 6.5 (7%) (295 K)
	1974 ± 5 (77 K)

a Emission decay times were monitored within the ^5^D_0_ → ^7^F_4_ (λ_em_ = 701.8 nm) and ^5^D_4_ → ^7^F_5_ (λ_em_ = 540.5 nm) transitions.

We calculated the thermometric parameters Δ and *S*_r_ (relative thermal sensitivity).^90^ The thermometric parameter Δ defined as *I*_Tb_/*I*_Eu_ allows for the conversion of the integrated intensity into temperature. Whereas *S*_r_ indicates the relative change in Δ per degree of temperature change and allows comparison of thermometric properties for different systems.^90^*S*_r_ is defined as
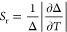


**Figure 10:**
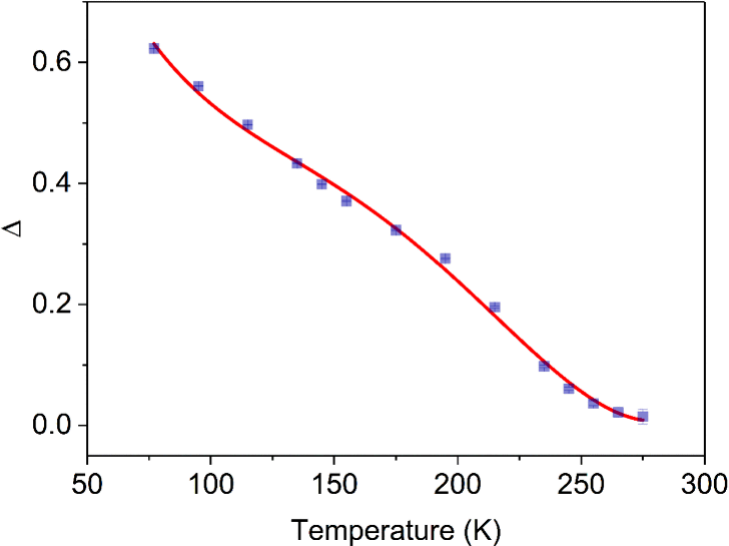
Temperature dependence of Δ for **[Eu**_**1.5**_**Tb**_**1.5**_**L**_**3**_**(μ**_**2**_**-F)**_**4**_**(NO**_**3**_**)**_**2**_**](NO**_**3**_**)**_**3**_**·3H**_**2**_**O**. The bars depict the errors in the thermometric parameters resulting from the propagation of the errors determined for *I*_Tb_ and *I*_Eu_. The red line represents the fitting curve and the goodness of fit is 0.9964.

The maximum value of *S*_r_ for a given temperature (*T*_m_) is marked as *S*_m_. *I*_Tb_ and *I*_Eu_ were calculated for the transitions ^5^D_4_ → ^7^F_5_ (range 533.95–564.25) and ^5^D_0_ → ^7^F_2_ (range 606.10–638.65) from spectra converted to cm^–1^. Figure 10 and Figure S27 present the temperature dependence Δ and the corresponding relative thermal sensitivities, respectively.

**[Eu**_**1.5**_**Tb**_**1.5**_**L**_**3**_**(μ**_**2**_**-F)**_**4**_**(NO**_**3**_**)**_**2**_**](NO**_**3**_**)**_**3**_**·3H**_**2**_**O** was found to exhibit ratiometric luminescence thermometer properties in the temperature range 77–275 K, with a maximum *S*_m_ of 0.46% K^–1^ at 222.1 K. The relative sensitivity is smaller than 1.0% K^–1^, which is an average result. Taking into account the spike in emission intensity for **[Tb**_**3**_**L**_**3**_**(μ**_**2**_**-F)**_**4**_**(NO**_**3**_**)**_**2**_**](NO**_**3**_**)**_**3**_**·3H**_**2**_**O** (Supporting Figures S17 and S19), the suppression of the weak temperature-dependent quenching of Eu^3+^ emission at 77 K and the increase in *S*_r_ values from about 100 K onward, it seems that the **[Eu**_**1.5**_**Tb**_**1.5**_**L**_**3**_**(μ**_**2**_**-F)**_**4**_**(NO**_**3**_**)**_**2**_**](NO**_**3**_**)**_**3**_**·3H**_**2**_**O** system will exhibit interesting thermometric properties in the cryogenic temperature range. Unfortunately, due to equipment limitations, we cannot measure emission in the 77–5 K range.

Emission, however, was not exhibited by the **[Dy**_**3**_**L**_**3**_**(μ**_**2**_**-F)**_**4**_**(NO**_**3**_**)**_**2**_**](NO**_**3**_**)**_**3**_**·2H**_**2**_**O** compound. The lack of luminescence is due to short Dy–Dy distances, which are responsible for the very efficient quenching process of Dy(III) emission by resonant cross-relaxation. Resonant cross-relaxation is possible due to the multiplicity of excited levels of the Dy(III) ion, for example, the energy of the ^4^F_9/2_ → ^6^H_5/2_ transition matches well with that of the ^6^H_15/2_ → ^6^H_5/2_ transition and is studied especially in various Dy(III) doped inorganic matrixes.^91,92^ To confirm the occurrence of the cross-relaxation process, we have recorded the emission spectrum for a corresponding mononuclear [DyL(NO_3_)_2_](NO_3_) complex (Supporting Figure S28). Dy(III) emission was observed previously for a similar mononuclear Dy(III) complex with the same hexaazamacrocyclic ligand.^44^

## Conclusions

In the triple-decker lanthanide(III) complexes of macrocycle L three metal ions are located in the centers of the hexaazamacrocycles, and the Ln(III) ions are linked by fluoride bridges. This type of structure differs from the well-known triple-decker porphyrin and phthalocyanine Ln(III) complexes^93,94^ or some nontetrapyrrolic macrocycles^95,96^ in which three macrocyclic units are linked by two Ln(III) ions positioned between them (Supporting Figure S29). The triple-decker Ln(III) complexes based on hexaazamacrocycle L also have different photophysical properties in comparison with tetrapyrrolic triple-deckers, where the extended aromatic system does not act as an efficient antenna for the luminescence of Eu(III). Similarly, the two types of complexes generate different crystal field, with a stronger axial field favoring SMM properties of Dy(III) in the case of hexazamacrocyles^31^ and a stronger equatorial field favoring SMM properties of Tb(III) in the case of phthalocyanines.^93^ The reactions of the mononuclear Ln(III) complexes of the macrocycle L derived from ethylenediamine and 2,6-diformylpyridiene with increasing amounts of fluoride lead to double-decker, triple-decker, or polymeric stacked complexes. In contrast, the linking of Ln(III) complexes of bulkier hexaazamacrocycles L1-L3 via two or three bridging fluoride anions appears to be limited to the formation of only double-decker derivatives.

The hexaazamacrocyclic imine ligand provides opportunities to construct trinuclear lanthanide complexes, in which nonradiative intramolecular energy transfer processes of the ligand → lanthanide and Ln–Ln types are present. The value of the energy of the ligand triplet state makes it possible to obtain a system with dual-center emission with the properties of a luminescent thermometer. The BET phenomenon is responsible for the strong temperature dependence of ^5^D_4_ Tb(III) luminescence, which is manifested by changes in the intensity and lifetime of the emission. At room temperature, ^5^D_0_ emission quenching processes are also present for compound **[Eu**_**3**_**L**_**3**_**(μ**_**2**_**-F)**_**4**_**(NO**_**3**_**)**_**2**_**](NO**_**3**_**)**_**3**_**·2H**_**2**_**O**, as evidenced by the values of *A*_nrad_ (330 s^–1^) and (38%). Due to the spike in Tb^3+^ emission intensity at 77 K as well as the suppression of ^5^D_0_ Eu^3+^ emission quenching and the increase in *S*_r_ values at low temperature, it is likely that the **Eu**_**1.5**_**Tb**_**1.5**_**L**_**3**_**(μ**_**2**_**-F)**_**4**_**(NO**_**3**_**)**_**2**_**](NO**_**3**_**)**_**3**_**·3H**_**2**_**O** system will exhibit interesting luminescent thermometer properties in a lower-temperature range of 5–80 K.

## Experimental Section

### Synthesis

The starting mononuclear [Ln(L)(NO_3_)_2_](NO_3_)·H_2_O complexes used for the synthesis of trinuclear derivatives have been obtained in a template condensation of 2,6-diformylpyridine, ethylenediamine and lanthanide(III) nitrate hydrates as reported previously.^40,97^

#### **[Tb_3_L_3_(μ_2_-F)_4_(NO_3_)_2_](NO_3_)_3_·_3_H_2_O**

[Tb(L)(NO_3_)_2_](NO_3_)·H_2_O (68.1 mg, 0,1 mmol) was stirred in the mixture of 15 mL of chloroform and 15 mL of methanol for 30 min, and then, 30.1 mg (0.18 mmol) of NEt_4_F·H_2_O was added with stirring. The clear solution was left to slowly evaporate to about 10 mL volume for 4 days. The formed [Tb_3_L_3_(μ_2_-F)_4_(NO_3_)_2_](NO_3_)_3_·5.2CHCl_3_·0.8CH_3_OH·H_2_O X-ray quality crystals were filtered and washed with 1 mL of methanol. These efflorescent crystals that rapidly lose organic crystallization solvents were dried to give a white powder and kept under ambient conditions. Yield 37 mg 49%. Anal. calcd for C_54_H_60_F_4_N_23_O_18_Tb_3_: C, 34.65; H, 3.23; N, 17.21. Found: C, 34.41; H, 3.45; N, 17.51.

The trinuclear Dy(III), Eu(III), Nd(III), and Y(III) complexes have been obtained in an analogous fashion. Similarly, the mixed **[Tb**_**1.5**_**Eu**_**1.5**_**L**_**3**_**(μ**_**2**_**-F)**_**4**_**(NO**_**3**_**)**_**2**_**](NO**_**3**_**)**_**3**_)**·3H**_**2**_**O** complex has been obtained starting from a mixture of 0.05 mmol of [Tb(L)(NO_3_)_2_](NO_3_)·H_2_O and 0.05 mmol of [EuL)(NO_3_)_2_](NO_3_)·H_2_O.

#### [Dy_3_L_3_(μ_2_-F)_4_(NO_3_)_2_](NO_3_)_3_·2H_2_O

Yield 37 mg 49%. Anal. calcd for C_54_H_58_Dy_3_F_4_N_23_O_17_: C, 34.78; H, 3.14; N, 17.28. Found: C, 34.57; H, 3.17; N, 17.20.

#### [Eu_3_L_3_(μ_2_-F)_4_(NO_3_)_2_](NO_3_)_3_·2H_2_O

Yield 37 mg 49%. Anal. calcd for C_54_H_58_Eu_3_F_4_N_23_O_17_: C, 35.38; H, 3.23; N, 17.21. Found: C, 35.75; H, 3.45; N, 17.51.

#### [Nd_3_L_3_(μ_2_-F)_4_(NO_3_)_2_](NO_3_)_3_·3H_2_O

Yield 40 mg 66%. Anal. calcd for C_54_H_60_F_4_N_23_Nd_3_O_18_: C, 35.48; H, 3.31; N, 17.57. Found: C, 35.19; H, 3.10; N, 17.58.

#### [Y_3_L_3_(μ_2_-F)_4_(NO_3_)_2_](NO_3_)_3_·3H_2_O

Yield 20 mg 36%. Anal. calcd for C_54_H_60_F_4_N_23_O_18_Y_3_: C, 39.03; H, 3.19; N, 19.38. Found: C, 39.33; H, 3.72; N, 19.00.

##### Isolation of the Crystals of Polymeric Complexes

[Nd(L)(NO_3_)_2_](NO_3_)·H_2_O (33.7 mg, 0.05 mmol) was combined with 18.4 mg (0.11 mmol) of NEt_4_F·H_2_O in the mixture of 5 mL of chloroform and 5 mL of methanol, and the mixture was left in a vial for 5 months. Partial evaporation of this sample resulted in the formation of single crystals of {[Nd_3_L_3_(μ_2_-F)_5_](NO_3_)_4_·2.5CH_3_OH·3H_2_O}_*n*_. Single crystals of the isomorphic Eu(III) complex were obtained in a similar manner. [Eu(L)(NO_3_)_2_](NO_3_)·H_2_O (33.3 mg, 0.05 mmol) was combined with 6.7 mg (0.1 mmol) of NEt_4_F·H_2_O in the mixture of 2.5 mL of chloroform and 2.5 mL of methanol. The mixture was left to evaporate to about 3 mL and filtered, and the filtrate was left to evaporate further until single crystals were formed.

##### Methods

The NMR spectra were recorded on a Bruker Avance 500 spectrometer. The elemental analyses were carried out on a PerkinElmer 2400 CHN and Elementar CHNS Vario EL Cube elemental analyzers. Magnetic measurements were carried out with Quantum Design MPMSXL–5 and MPMS3 SQUID magnetometers. The direct current (dc) magnetic susceptibility measurements were carried out in the temperature range of 1.8–300 K with applied magnetic fields of 1000 Oe. The alternating current (ac) susceptibility measurements in zero dc field were performed with an oscillating magnetic field of 3 Oe at frequencies ranging from 1 to 960 Hz. Background corrections for the sample holder and diamagnetic contribution estimated from the Pascal constants were applied.

X-ray diffraction data for the crystals were collected at 100 K on a κ-geometry Rigaku XtaLAB Synergy-DW diffractometer (ω scans) with Cu Kα radiation and a rotating-anode X-ray source. Data collections, cell refinements, data reductions, and analyses, including absorption corrections, were carried out with *CrysAlisPRO*.^98^ With the use of Olex2,^99^ structures were solved with the *SHELXT* program^100^ employing dual-space algorithm and refined on *F*^2^ by a full-matrix least-squares procedure using *SHELXL*(101) with anisotropic displacement parameters for the fully occupied non-H atoms. Some positions of partially occupied atoms were also refined anisotropically. Figures presenting the molecular structures were made using the Mercury^102^ or DIAMOND^103^ program. Details of structure determination together with the Supporting Table S1 are provided in the Supporting Information, and the crystallographic information files (CIFs) were deposited at the Cambridge Crystallographic Data Centre (CCDC nos. 2325397–2325399)

Emission and excitation spectra were recorded with an Edinburgh Instruments FLSP 920 spectrofluorimeter equipped with a 450 W xenon lamp and an R13456 Hamamatsu photomultiplier. These spectra were corrected for the instrument response. Emission decay curves were measured using a μF920H 60W Xe flashlamp (Edinburgh Instruments Ltd.) and analyzed with a program coupled to the spectrofluorimeter. The temperature-dependent photoluminescence measurements were performed using an OptistatDN cryostat from Oxford Instruments (nitrogen bath cryostat, sample in exchange gas). The temperature was controlled by an Intelligent Temperature Controller (ITC-601PT Oxford) and was stabilized 10 min before each measurement. The absolute overall emission quantum yield of **[Eu**_**3**_**L**_**3**_**(μ**_**2**_**-F)**_**4**_**(NO**_**3**_**)**_**2**_**](NO**_**3**_**)**_**3**_**·2H**_**2**_**O** was measured at room temperature using an integrating sphere of 120 mm in diameter (Edinburgh Instruments). Details of luminescence studies are provided in the Supporting Information.

## Supporting Information Available

Details of structure refinement and luminescence studies, Supporting Tables S1 and S2, and Supporting Figures S1–S29 (views of crystal structures, magnetic and luminescence data) (PDF)
