# Supplementary material for: Triple-Decker Hexaazamacrocyclic Lanthanide(III) Complexes: Structure, Magnetic Properties, and Temperature-Dependent Luminescence
Source: Inorg Chem. 2024 Aug 9;63(34):15875–87. doi: 10.1021/acs.inorgchem.4c02047 (PMC11351181; doi:10.1021/acs.inorgchem.4c02047)
Supplement: Supplementary file 1 — ic4c02047_si_001.pdf [file ic4c02047_si_001.pdf]

Supporting Information

Triple-Decker Hexaazamacrocyclic Lanthanide(III) Complexes: Structure, Magnetic Properties and Temperature Dependent Luminescence.

Paula Gawryszewska, Katarzyna Ślepokura and Jerzy Lisowski *

Department of Chemistry, University of Wrocław, 14 F. Joliot-Curie, 50-383 Wrocław, Poland.

E-mail: jerzy.lisowski@chem.uni.wroc.pl

Contents

X-ray crystallography……………………………..…………………………………S2-S4

Lumienescence…………………………………………………………………… S5-S6

Supporting crystal structure figures……………………………………………….S6-S12

Magnetic data figures…………………………………………………..………S13-S17

Supporting luminescence figures…………………………………………………S18-S26

Comparison of triple-decker Ln(III) complexes…………………………………..…S27

References……………………………………………………..………..………S27-S28

X-ray crystallography

Crystals of $[DyL(NO_3)_2](NO_3)$ are isomorphous with the previously published yttrium compound (Starynowicz, P.; Lisowski, J. *Polyhedron* **2015**, *85*, 232-238.). Therefore for the refinement of the structure of Dy(III) crystal, the atomic coordinates taken from Y(III) crystal were used. The model was then shifted by a vector (0, 0, -0.5).

H atoms in all the crystal structures were found in the difference Fourier maps or were included using geometrical considerations. In the final refinement cycles, all C-bound and the methanol hydroxyl H atoms were repositioned in their calculated positions and were refined using a riding model, with C–H = 0.95-1.00 Å and O–H = 0.84 Å, and with $U_{iso}(H) = 1.2U_{eq}(C)$ for CH, CH_2 or $1.5U_{eq}(O,C)$ for OH, CH_3. O1W and O2W water H atoms in the crystal of polymeric $\{[Nd_3L_3(\mu_2\text{-}F)_5](NO_3)_4\cdot 2.5CH_3OH\cdot 3H_2O\}_n$ compound were refined with O–H and H···H distances restrained to 0.840(2) and 1.360(2) Å, respectively, and with $U_{iso}(H) = 1.5U_{eq}(O)$. In the final cycles, they were constrained to ride on their parent atoms (AFIX 3 instruction in *SHELXL*). Remaining water H atoms (site occupation factors, SOFs < 0.5) were not found in difference Fourier maps.

All solvent ($CHCl_3$, methanol and water) molecules, as well as non-coordinated NO_3^- anions in the crystals of the trimeric and polymeric compounds, $[Tb_3L_3(\mu_2\text{-}F)_4(NO_3)_2](NO_3)_3\cdot 5.2CHCl_3\cdot 0.8CH_3OH\cdot H_2O$ and $\{[Nd_3L_3(\mu_2\text{-}F)_5](NO_3)_4\cdot 2.5CH_3OH\cdot 3H_2O\}_n$, were found to be disordered. (One of the nitrate ions in the trimeric compound crystal was disordered about an inversion centre). They were refined (mainly anisotropically) in one, partially occupied position, or in two positions. In the refinement procedures, some geometrical restraints (DFIX for methanol molecules, SADI for $CHCl_3$ molecules, SAME for NO_3^- and $CHCl_3$), and restraints on anisotropic displacement parameters (SIMU, ISOR) were applied to get acceptable and appropriate models of the disordered regions. Due to disorder in the crystals of the trimeric and polymeric compounds, their formulae should be treated as approximations.

Supporting Table S1. Crystallographic data for $[DyL(NO_3)_2](NO_3)$, $[Tb_3L_3(\mu_2\text{-}F)_4(NO_3)_2](NO_3)_3\cdot5.2CHCl_3\cdot0.8CH_3OH\cdot H_2O$ and $\{[Nd_3L_3(\mu_2\text{-}F)_5](NO_3)_4\cdot2.5CH_3OH\cdot3H_2O\}_n$

	$[DyL(NO_3)_2](NO_3)$	$[Tb_3L_3(\mu_2\text{-}F)_4(NO_3)_2](NO_3)_3\cdot5.2CHCl_3\cdot0.8CH_3OH\cdot H_2O$	$\{[Nd_3L_3(\mu_2\text{-}F)_5](NO_3)_4\cdot2.5CH_3OH\cdot3H_2O\}_n$
CCDC No.	2325397	2325398	2325399
Chemical formula	$C_{18}H_{18}DyN_9O_9$	$C_{60}H_{64.4}Cl_{15.6}F_4N_{23}O_{16.8}Tb_3$	$C_{56.5}H_{70}F_5N_{22}Nd_3O_{17.5}$
M_r	666.91	2482.32	1865.06
Crystal system, space group	Monoclinic, $P2_1/n$	Orthorhombic, *Pbcn*	Monoclinic, $C2/c$
Temperature (K)	100	100	100
a, b, c (Å)	14.0860(11), 11.3089(10), 14.2395(12)	22.510(3), 21.345(3), 18.738(3)	24.828(4), 12.345(2), 24.880(4)
α, β, γ (°)	90, 95.76(2), 90	90, 90, 90	90, 113.02(2), 90
V (Å^3)	2256.9 (3)	9003 (2)	7019 (2)
Z	4	4	4
Radiation type	Cu $K\alpha$	Cu $K\alpha$	Cu $K\alpha$
μ (mm^{-1})	18.37	16.35	17.51
Crystal size (mm)	0.22 × 0.21 × 0.16	0.12 × 0.11 × 0.08	0.29 × 0.04 × 0.02
Diffractometer	Rigaku XtaLAB Synergy-DW	Rigaku XtaLAB Synergy-DW	Rigaku XtaLAB Synergy-DW
Absorption correction	Multi-scan	Analytical	Gaussian
T_{min}, T_{max}	0.147, 1.000	0.245, 0.416	0.074, 1.000
No. of measured, independent and observed [$I > 2\sigma(I)$] reflections	93272, 4660, 4571	36755, 8822, 7425	20310, 6641, 5658
R_{int}	0.076	0.032	0.033
$(\sin \theta/\lambda)_{max}$ (Å^{-1})	0.628	0.621	0.620
$R[F^2 > 2\sigma(F^2)]$, $wR(F^2)$, S	0.033, 0.093, 1.07	0.039, 0.111, 1.05	0.040, 0.111, 1.07
No. of reflections	4660	8822	6641
No. of parameters	335	755	533
No. of restraints	0	333	86
H-atom treatment	H-atom parameters constrained	H-atom parameters constrained	H-atom parameters constrained
$\Delta\rho_{max}$, $\Delta\rho_{min}$ (e Å^{-3})	1.26, −1.61	0.84, −0.88	1.04, −1.42

Computer programs: *CrysAlis PRO* (Rigaku OD, 2020; 2023), *SHELXT-2014* (Sheldrick, 2015), *SHELXL2014/7* (Sheldrick, 2015).

Supporting Table S2. Continuous Shape Measurements (CShMs) for Tb^{III} ions in TbL_{10} polyhedra in the trimeric cation $[Tb_3L_3(\mu_2\text{-}F)_4(NO_3)_2]^{3+}$ present in the crystal of $[Tb_3L_3(\mu_2\text{-}F)_4(NO_3)_2](NO_3)_3\cdot 5.2CHCl_3\cdot 0.8CH_3OH\cdot H_2O$. The smallest deviations from the ideal coordination geometries are marked in grey.

TbL_{10}	HD	TD	SDD	JSPC	JATDI	JMBIC	JBCSAPR	JBCCU	PAPR	PPR	OBPY	EPY	DP
Tb1	7.357	2.007	2.391	2.311	19.010	6.950	3.848	9.594	11.103	10.915	15.713	24.155	36.994
Tb2	8.174	2.445	2.145	2.444	19.768	7.151	3.743	10.260	10.578	10.843	16.326	23.240	34.968

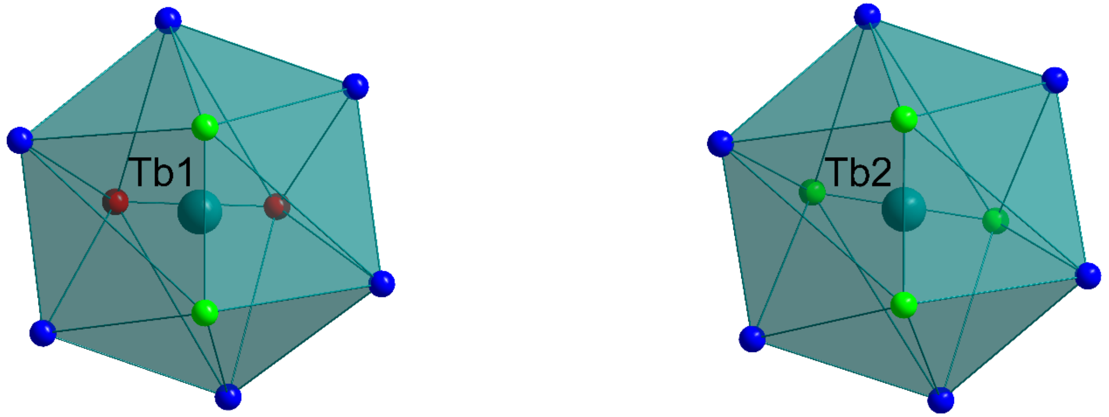

Ideal structures for ML_{10}: HD (D_{4h}) – Hexadecahedron (2:6:2) or (1:4:4:1); TD (C_{2v}) – Tetradecahedron (2:6:2); SDD (D_2) – Staggered Dodecahedron (2:6:2); JSPC (C_{2v}) – Sphenocorona J87; JATDI (C_{3v}) – Augmented tridiminished icosahedron J64; JMBIC (C_{2v}) – Metabidiminished icosahedron J62; JBCSAPR (D_{4d}) – Bicapped square antiprism J17; JBCCU (D_{4h}) – Bicapped cube J15; PAPR (D_{5d}) – Pentagonal antiprism; PPR (D_{5h}) – Pentagonal prism; OBPY (D_{8h}) – Octagonal bipyramid; EPY (C_{9v}) – Enneagonal pyramid; DP (D_{10h}) – Decagon.

Luminescence

Descriptions of radiative (A_{rad}) and non-radiative (A_{nrad}) decay rates, intrinsic emission quantum yield (Q_{Eu}^{Eu}), sensitization efficiency (η), as well as $\frac{\delta\Delta}{\Delta}$ calculations are provided below.

Based on the emission spectrum of the **$[Eu_3L_3(\mu_2\text{-}F)_4(NO_3)_2](NO_3)_3\cdot 2H_2O$** complex , the radiative (A_{rad}) and non-radiative (A_{nrad}) rates were determined from the coefficients of spontaneous emission ($A_{0\lambda}$). The spontaneous emission coefficients (also called the Einstein coefficient for spontaneous emission or probability for spontaneous emission) were calculated by taking the magnetic dipole transition $^5D_0 \rightarrow {}^7F_1$ as the reference, as this transition is practically insensitive to the chemical environment around Eu^{3+}. The following equation was used [1]:

$$A_{0\lambda} = A_{01}\left(\frac{S_{0\lambda}}{S_{01}}\right)\left(\frac{\sigma_{01}}{\sigma_{0\lambda}}\right) \qquad (1)$$

where S_{01} and $S_{0\lambda}$ are the areas of the bands corresponding to the $^5D_0 \rightarrow {}^7F_1$ and $^5D_0 \rightarrow {}^7F_\lambda$ transitions, with σ_{01} and $\sigma_{0\lambda}$ being their energy barycenters, respectively. A_{01}, in equation (1), is given by the relation $A_{01} = 0.31 \cdot 10^{-11}((n^3)(\sigma_{01})^3$. The value of the refractive index was taken as 1.5, the value used for coordination compounds of lanthanides with organic ligands [2]. Nm in the emission spectrum were converted to cm^{-1} using Jacobian transformation [3].

All emission probabilities $A_{0\lambda}$ (λ = 1, 2 and 4)that depopulate $^5D_0$ level were used to determined A_{rad}

$$A_{rad} = \sum A_{0\lambda} \qquad (2)$$

$$\tau_{rad} = \frac{1}{A_{rad}} \qquad (3)$$

were τ_{rad} is radiative lifetime.

$$A_{nrad} = A - A_{rad} \qquad (4)$$

were $A = \frac{1}{\tau}$ and τ is $^5D_0$ experimental lifetime for **$[Eu_3L_3(\mu_2\text{-}F)_4(NO_3)_2](NO_3)_3\cdot 2H_2O$** (with direct Eu^{3+} excitation, λ_{exc} = 465 nm)

The intrinsic emission quantum yield is defined as [4]:

$$Q_{Eu}^{Eu} = \frac{A_{rad}}{A_{nrad}+A_{rad}} = \frac{\tau}{\tau_{rad}} \qquad (5)$$

The method for estimating the uncertainty of thermometric parameters is given below.

$\frac{\delta\Delta}{\Delta}$ was estimated as [5]:

$$\frac{\delta\Delta}{\Delta} = \sqrt{\left(\frac{\delta I_1}{I_1}\right)^2 + \left(\frac{\delta I_2}{I_2}\right)^2} \qquad (6)$$

where $\frac{\delta I_1}{I_1}$ and $\frac{\delta I_1}{I_1}$ are uncertainties on I_1 (integral intensity of the $^5D_4 \rightarrow {}^7F_5$ band) and I_2 (integral intensity of the $^5D_0 \rightarrow {}^7F_2$ band). $\frac{\delta I_1}{I_1}$ and $\frac{\delta I_1}{I_1}$were estimated using the SNR values (signal to noise ratio) dividing the readout fluctuations of the baseline by the maximum intensity of $^5D_4 \rightarrow {}^7F_5$ or $^5D_0 \rightarrow {}^7F_2$

transition. The dependence of the Δ parameter on temperature was fitted using a fourth-degree polynomial function. Higher degree polynomial functions provided better fit, but were not used due to the lack of physical meaning.

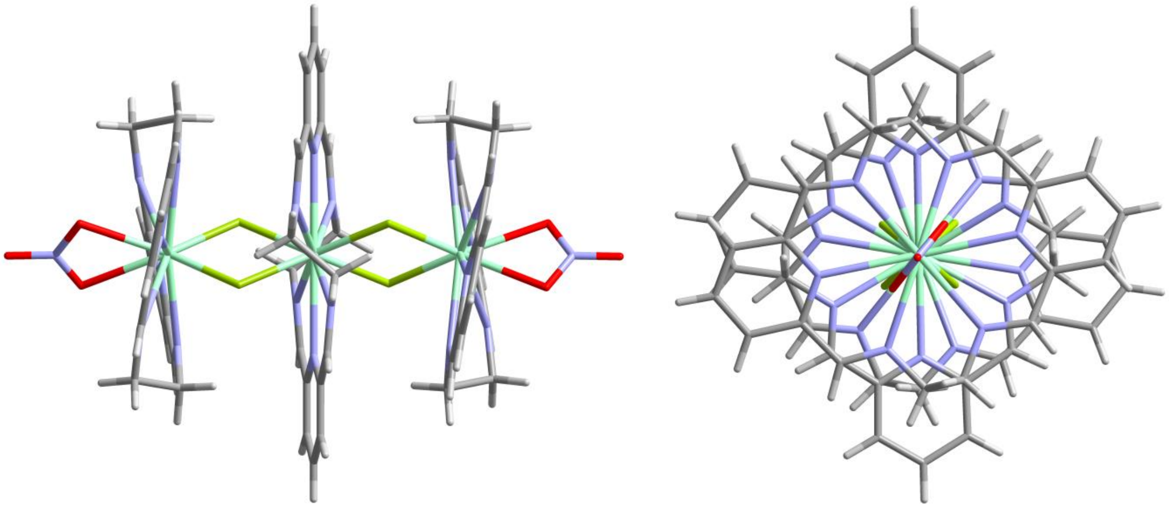

Supporting Figure S1. Side and top view of the trinuclear Dy(III) complex cation. Colour code: Dy, green; F, lime; O, red; N, blue; C, grey; H, light grey.

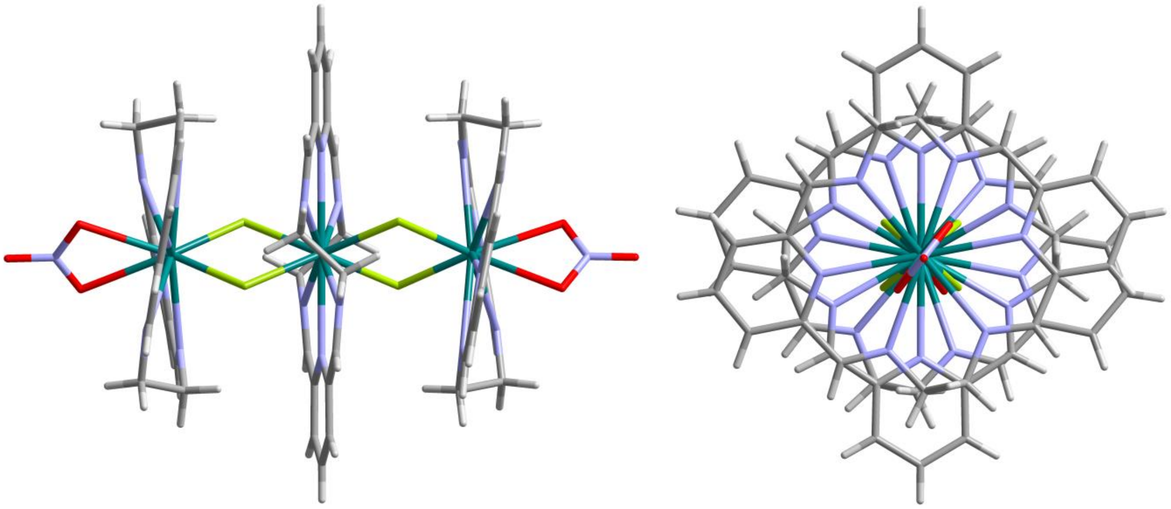

Supporting Figure S2. Side and top view of the trinuclear Nd(III) complex cation. Colour code: Nd, teal; F, lime; O, red; N, blue; C, grey; H, light grey.

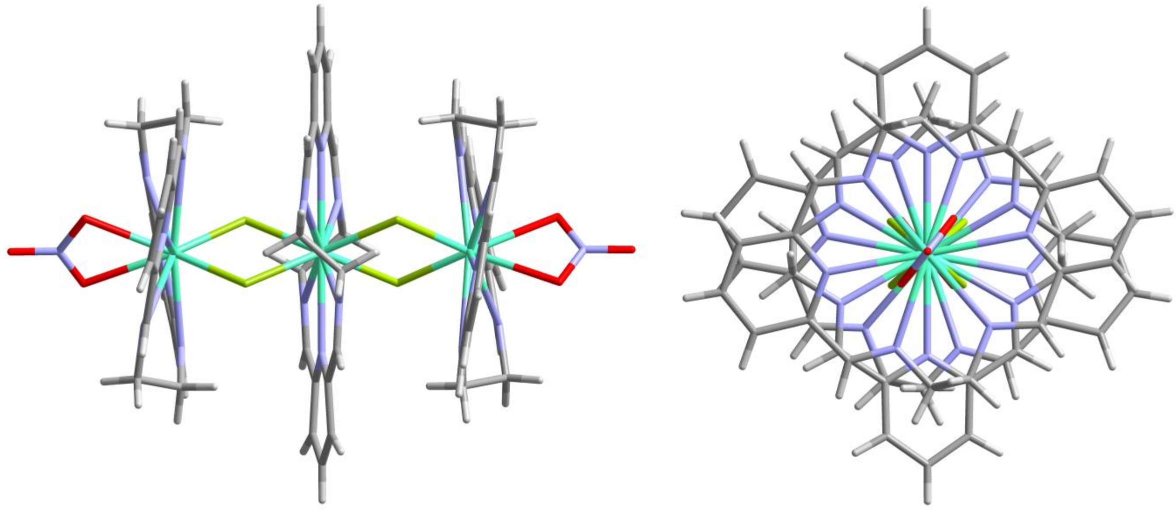

Supporting Figure S3. Side and top view of the trinuclear Eu(III) complex cation. Colour code: Eu, green; F, lime; O, red; N, blue; C, grey; H, light grey.

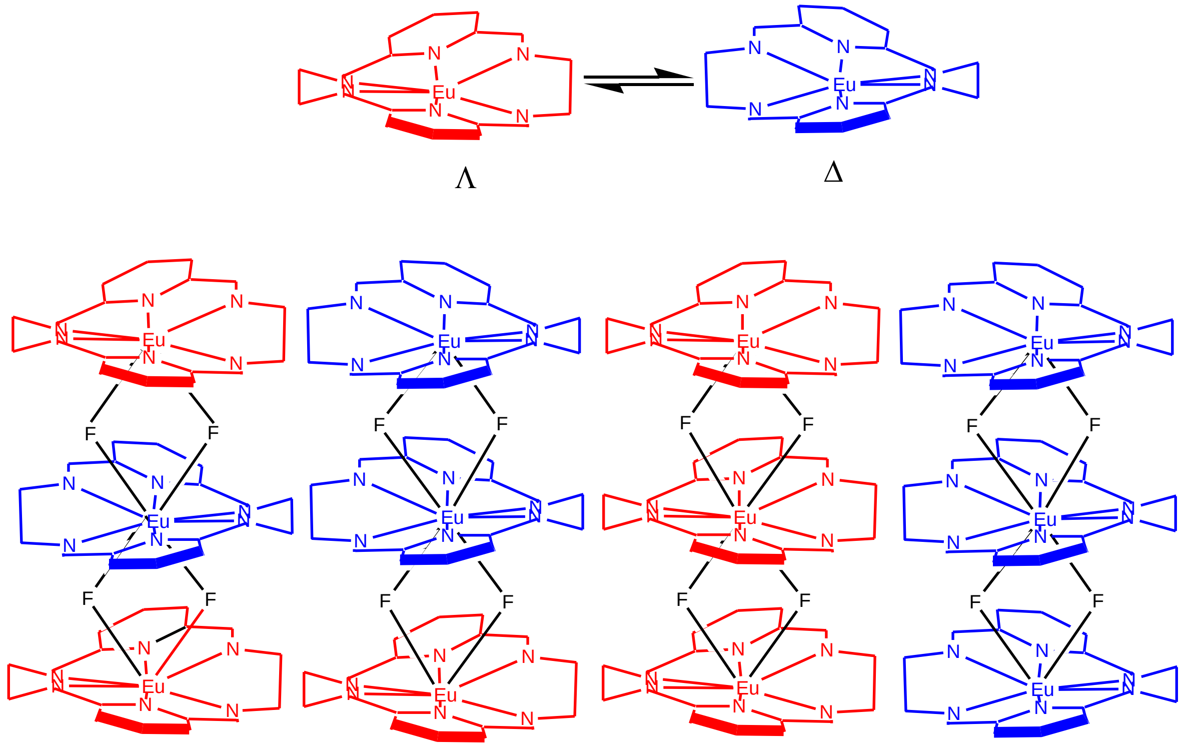

heterochiral triplexes (not observed)

racemic homochiral triplexes observed in the crystal structure

Supporting Figure S4. Top: helical conformation of monomeric Ln(III) complexes of L (the enantiomeric complex cation molecules differ in mutual orientation of pyridine rings). Right: Side views of ΔΔΔ (blue) and ΛΛΛ (red) trimeric Ln(III) complex cations observed in the discussed crystal structures. Left: a putative mixed heterochiral Δ/Λ trimers. Axial nitrate anions and charge are omitted for simplicity. Note that in this figure all the macrocyclic units are oriented in the same way along the main axis for clarity, in the real structure the middle macrocycle is rotated at ca. right angle with respect to the outer macrocyclic units (see Supporting Figure S5).

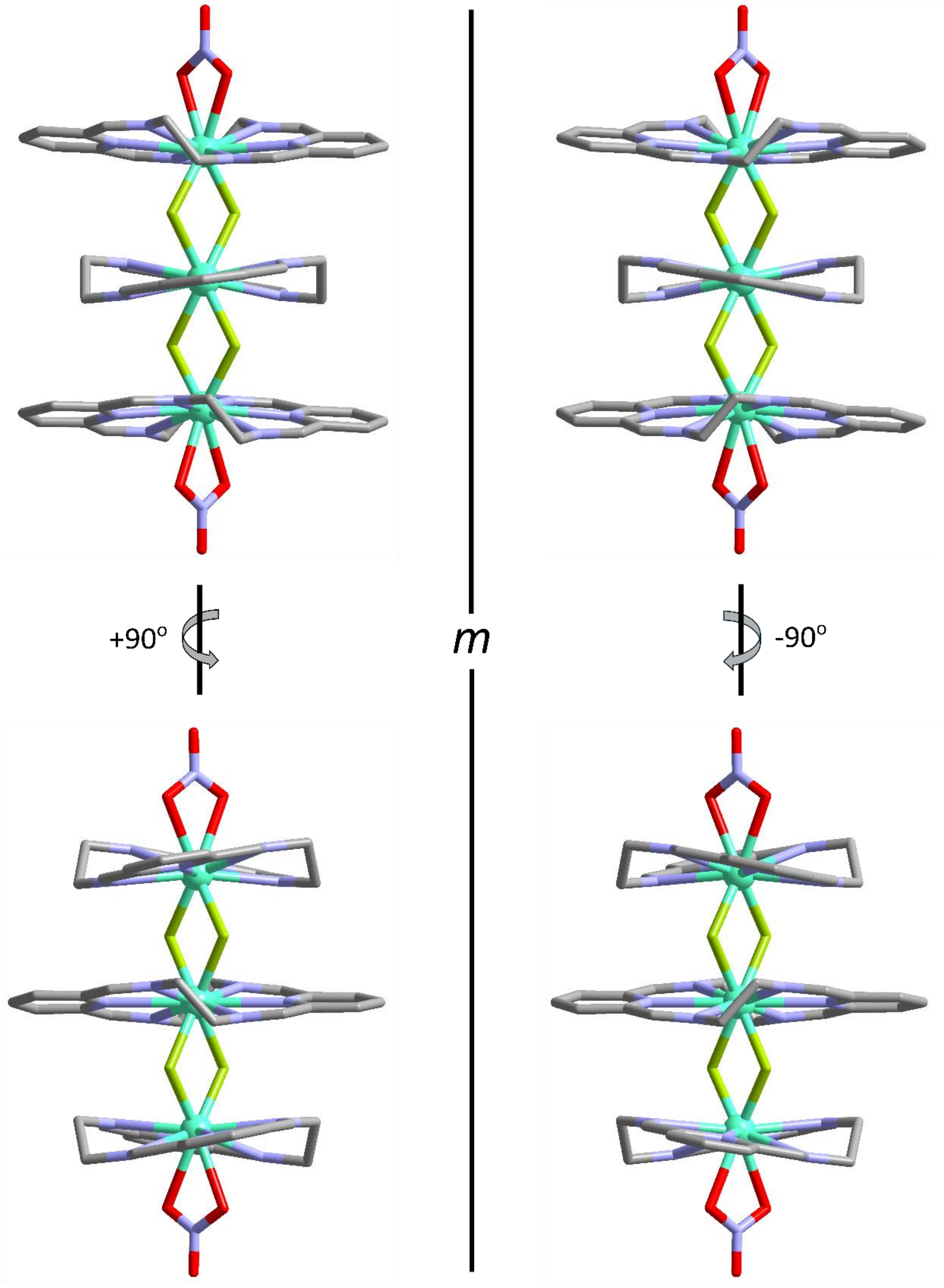

ΔΔΔ **ΛΛΛ**

Supporting Figure S5. Side views of ΔΔΔ (left) and ΛΛΛ (right) enantiomers of trimeric Tb(III) complex cations observed in the racemic crystal. For each enantiomer two views corresponding to a 90 degree rotation along the main axis are presented (top and bottom). Colour code: Tb, green; F, lime; O, red; N, blue; C, grey.

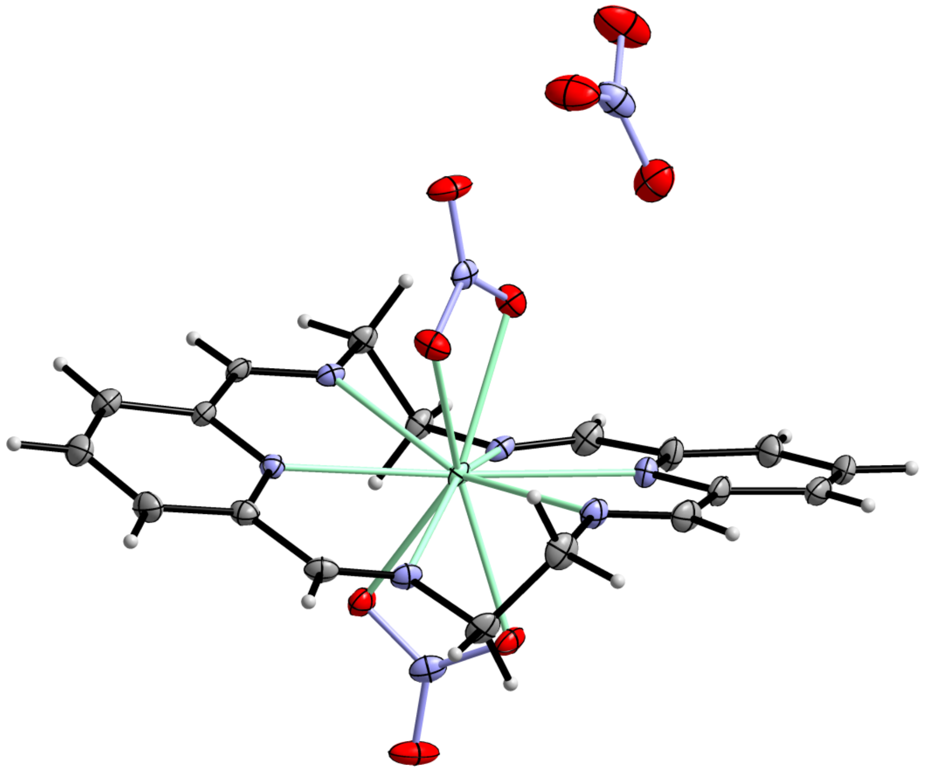

Supporting Figure S6. X-ray structure of the mononuclear [DyL(NO_3)$_2$](NO_3) complex. Displacement ellipsoids are shown at 50% probability level. Colour code: Dy, green; O, red; N, blue; C, grey; H, light grey.

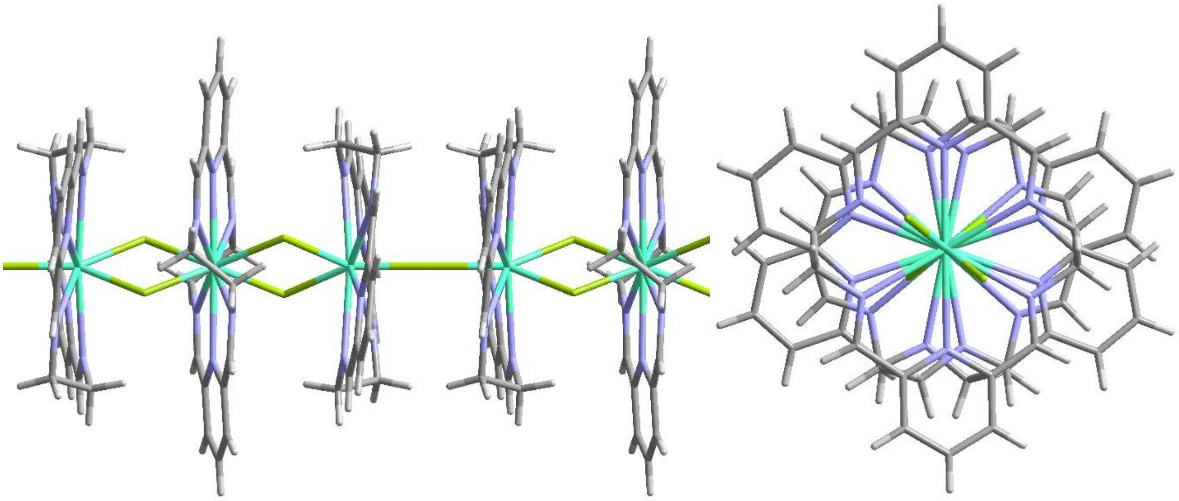

Supporting Figure S7. Side and top view of the polymeric Eu(III) complex cation. Colour code: Eu, green; F, lime; N, blue; C, grey; H, light grey.

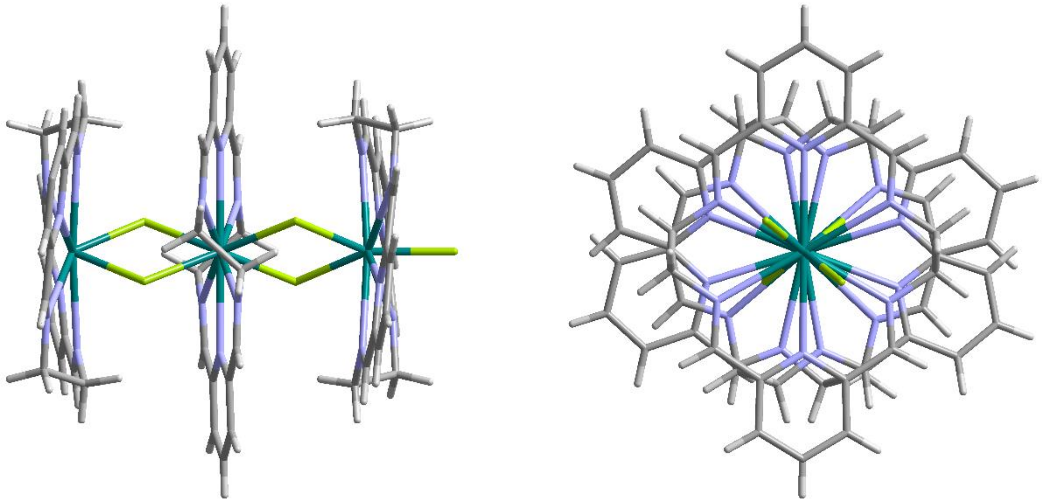

Supporting Figure S8. Side and top view of the trinuclear $[Nd_3L_3(\mu_2\text{-}F)_5]^{4+}$ unit forming the polymeric Nd(III) complex. Colour code: Nd, teal; F, lime; N, blue; C, grey; H, light grey.

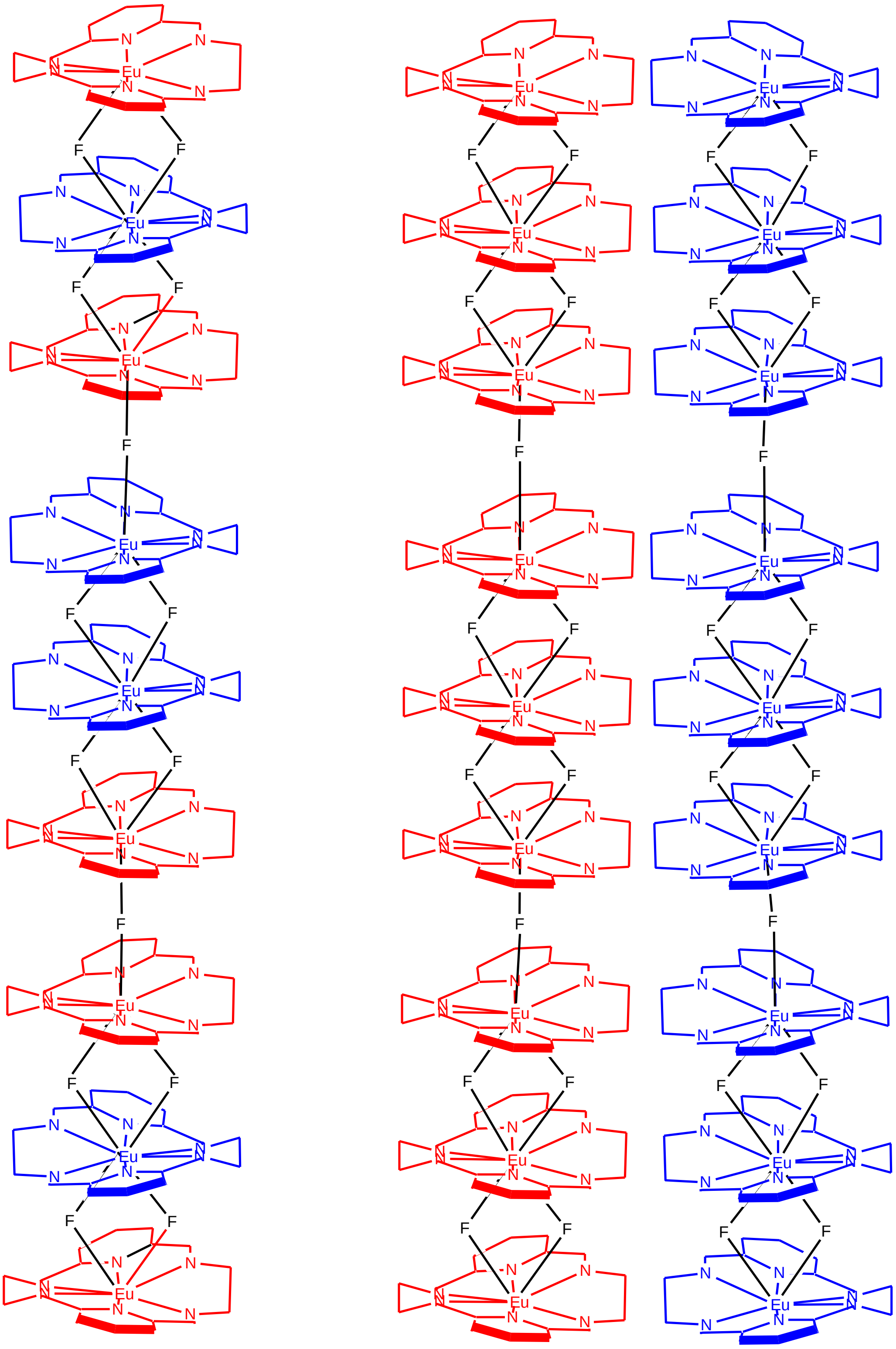

random heterochiral polymer (not observed)

racemic homochiral polymer observed in the crystal structure

Supporting Figure S9. Right: Side views of all-Δ (blue) and all-Λ (red) polymeric Ln(III) complex cations observed in the discussed crystal structures. Left: a putative mixed heterochiral Δ/Λ polymer. Note that in this simplified picture all the macrocyclic units are oriented in the same way along the polymer axis for clarity.

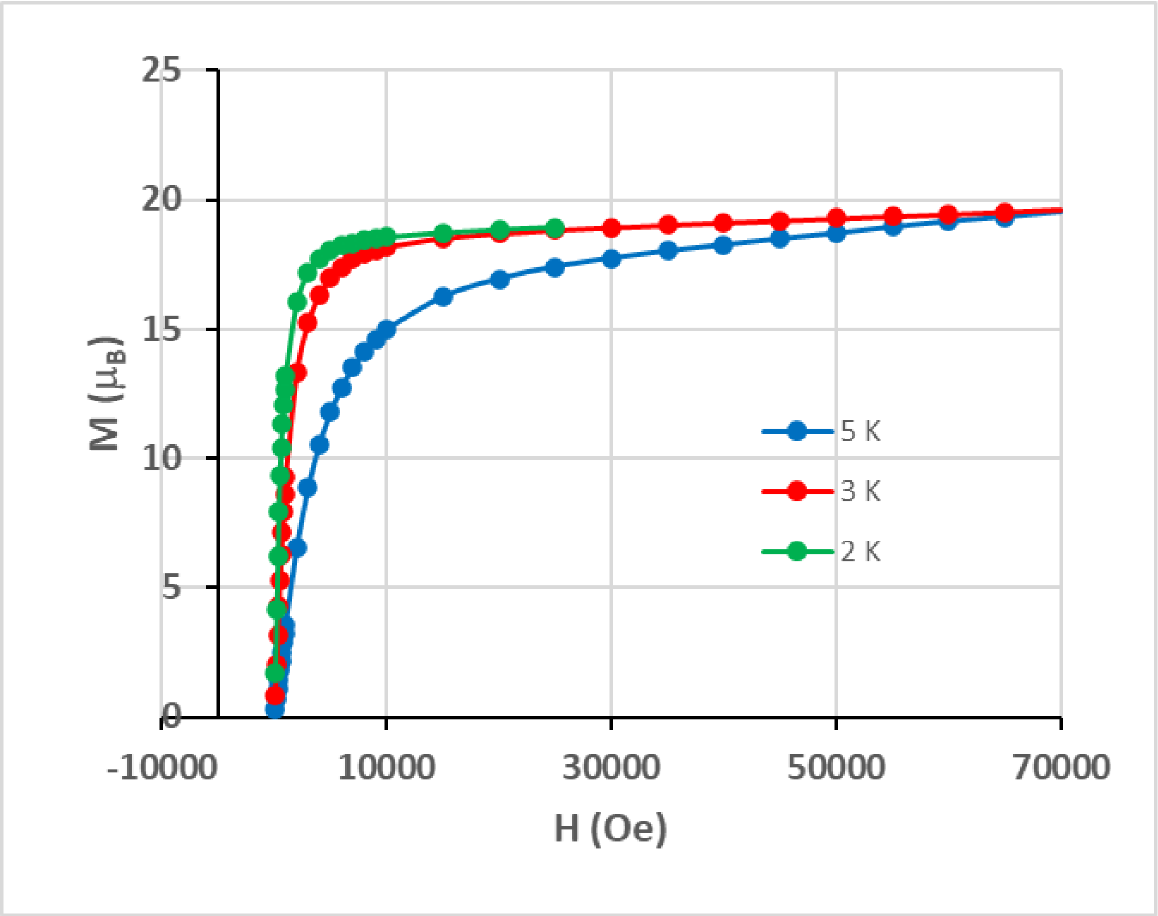

Supporting Figure S10. Field dependence of the magnetization *M* vs *H* at 2, 3 and 5 K for the trinuclear Dy(III) complex **$[Dy_3L_3(\mu_2\text{-}F)_4(NO_3)_2](NO_3)_3\cdot 2H_2O$** (the solid lines are eye guides only).

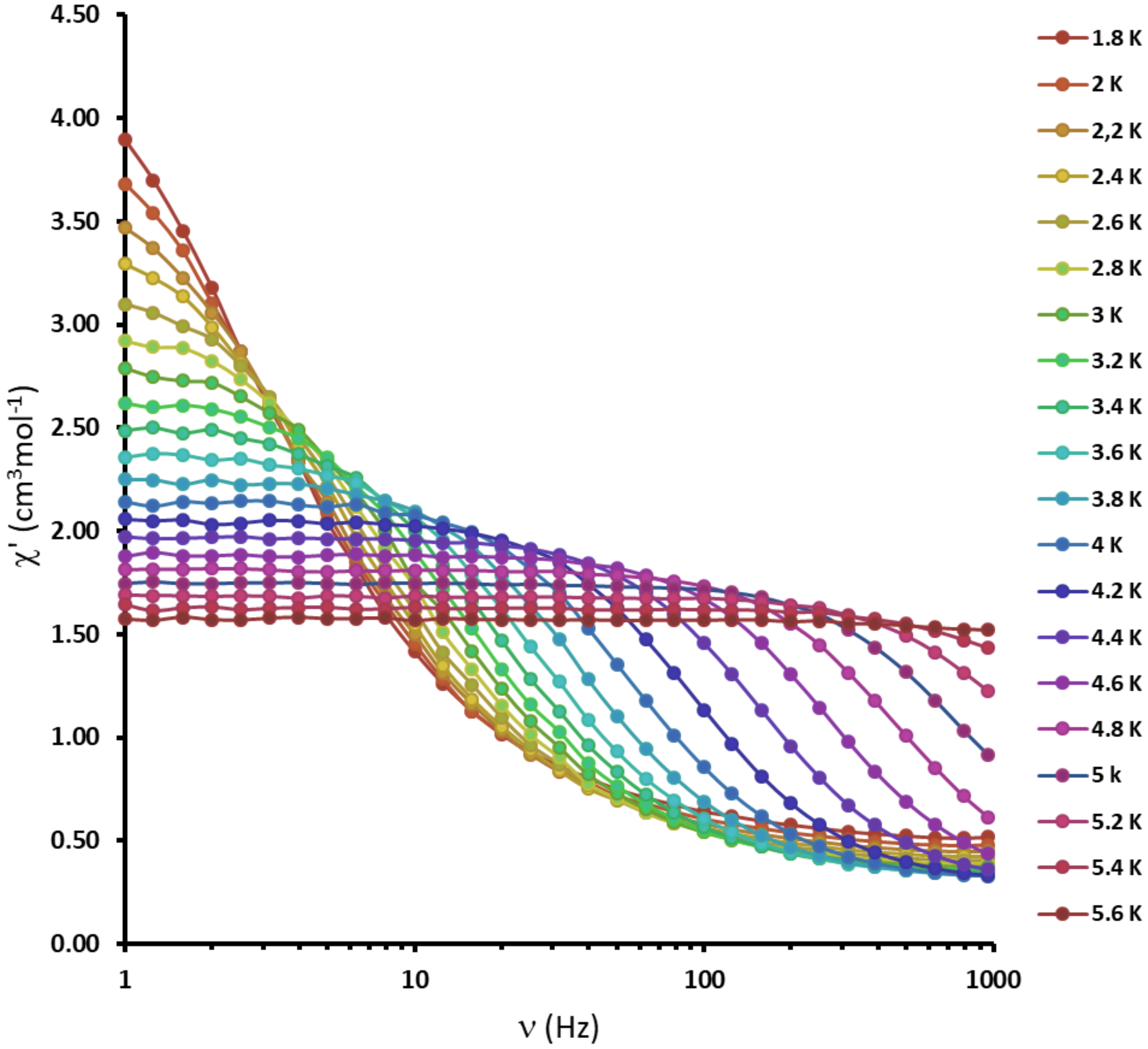

Supporting Figure S11. Frequency dependence of the real component of ac susceptibility (χ') for the trinuclear Dy(III) complex **$[Dy_3L_3(\mu_2\text{-}F)_4(NO_3)_2](NO_3)_3\cdot 2H_2O$** under zero dc field at ac frequencies of 1-960 Hz in the temperature range of 1.8 to 5.6 K.

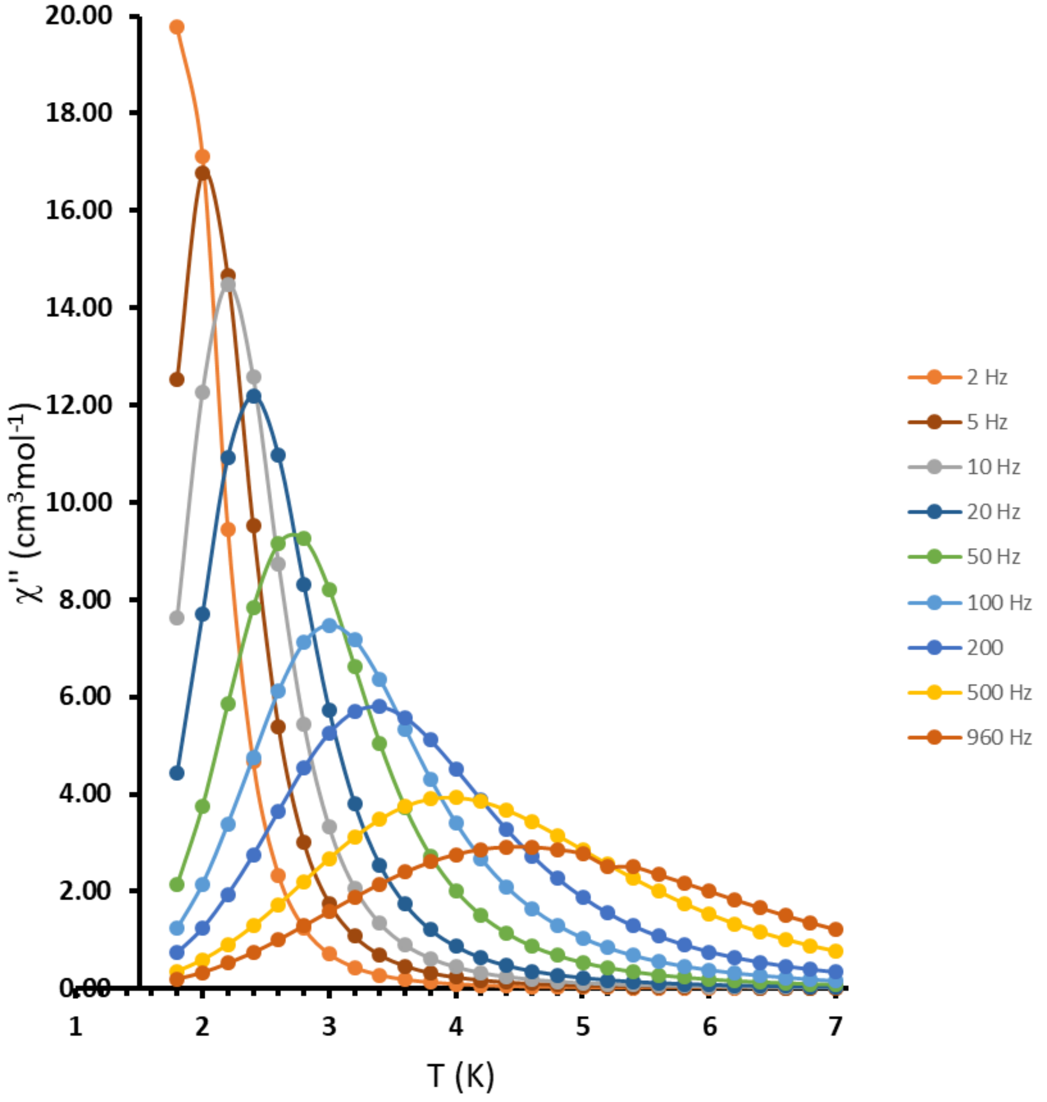

Supporting Figure S12. Temperature dependence of the imaginary component of ac susceptibility (χ'') for the trinuclear Dy(III) complex **$[Dy_3L_3(\mu_2\text{-}F)_4(NO_3)_2](NO_3)_3\cdot 2H_2O$** under zero dc field.

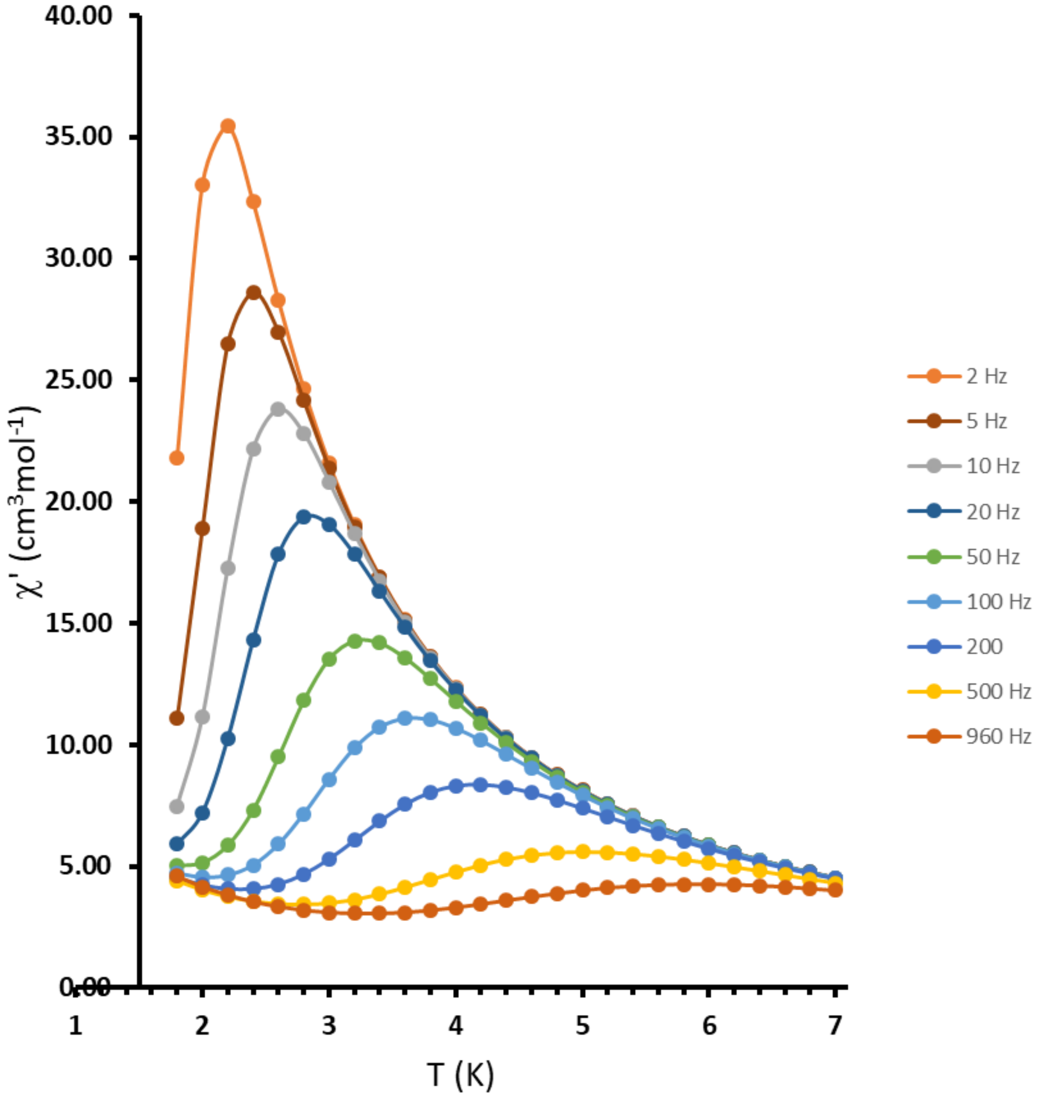

Supporting Figure S13. Temperature dependence of the real component of ac susceptibility (χ') for the trinuclear Dy(III) complex **$[Dy_3L_3(\mu_2\text{-}F)_4(NO_3)_2](NO_3)_3\cdot 2H_2O$** under zero dc field.

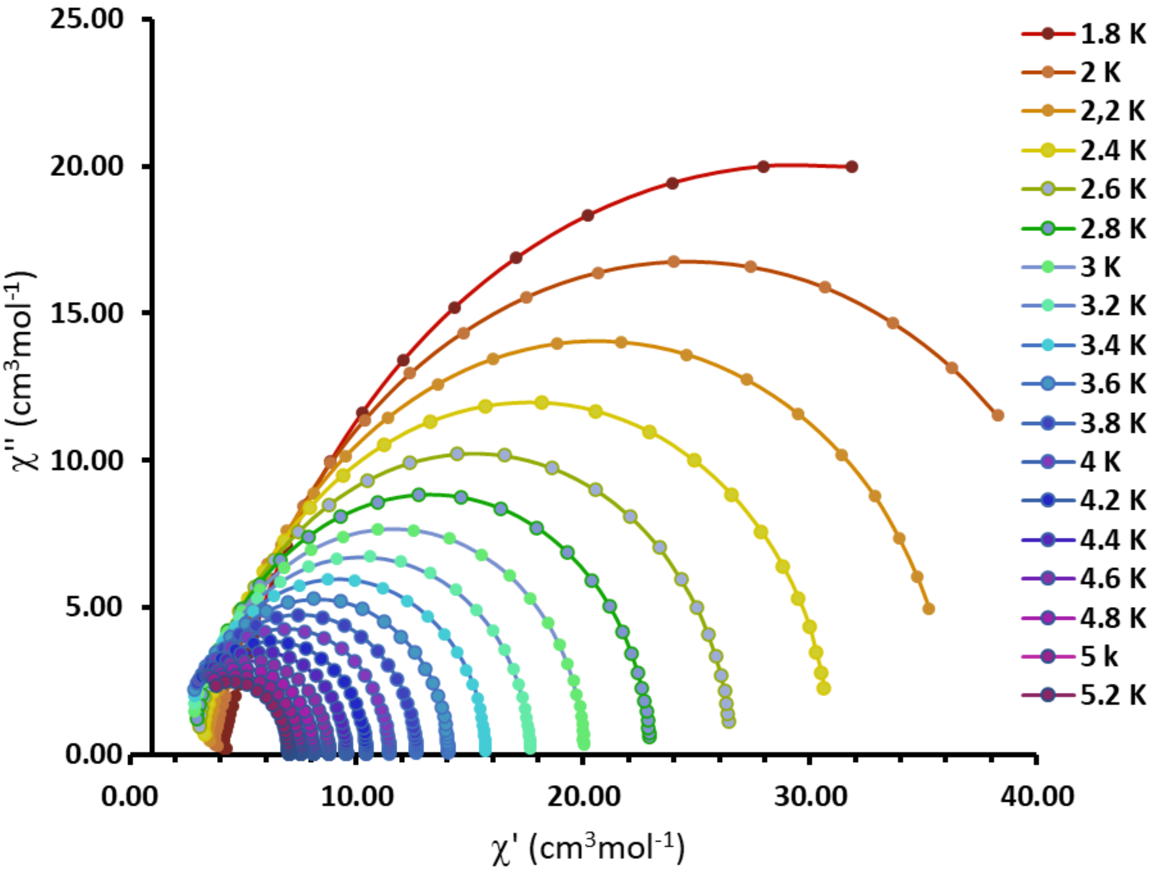

Supporting Figure S14. Cole-Cole plots of trinuclear Dy(III) complex **$[Dy_3L_3(\mu_2\text{-}F)_4(NO_3)_2](NO_3)_3\cdot2H_2O$** .

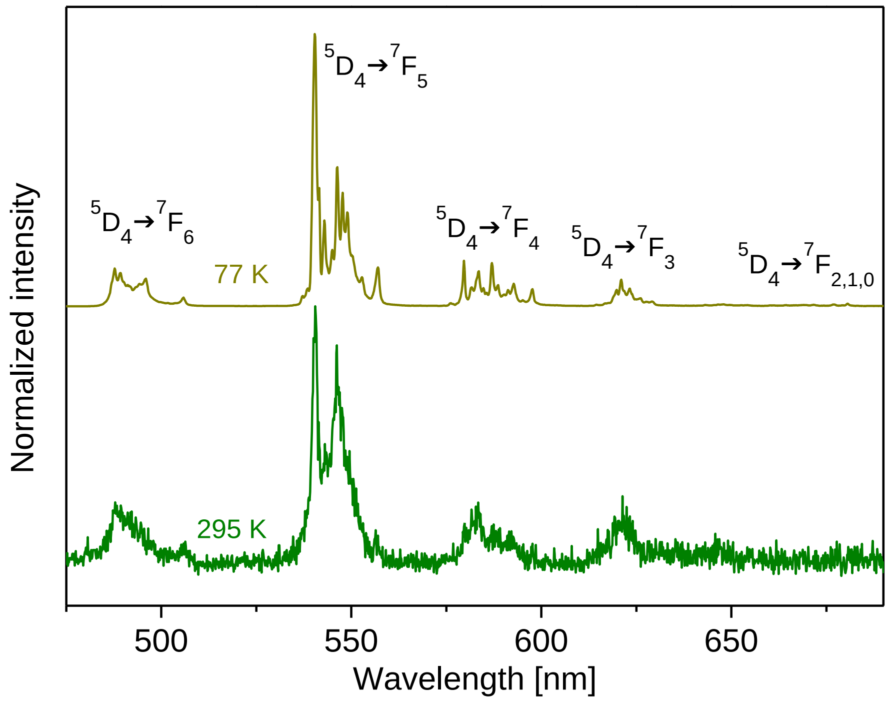

Supporting Figure S15. Emission spectra of single crystals of **$[Tb_3L_3(\mu_2\text{-}F)_4(NO_3)_2](NO_3)_3$** at 295 and 77 K, λ_{exc} = 337 nm.

a)

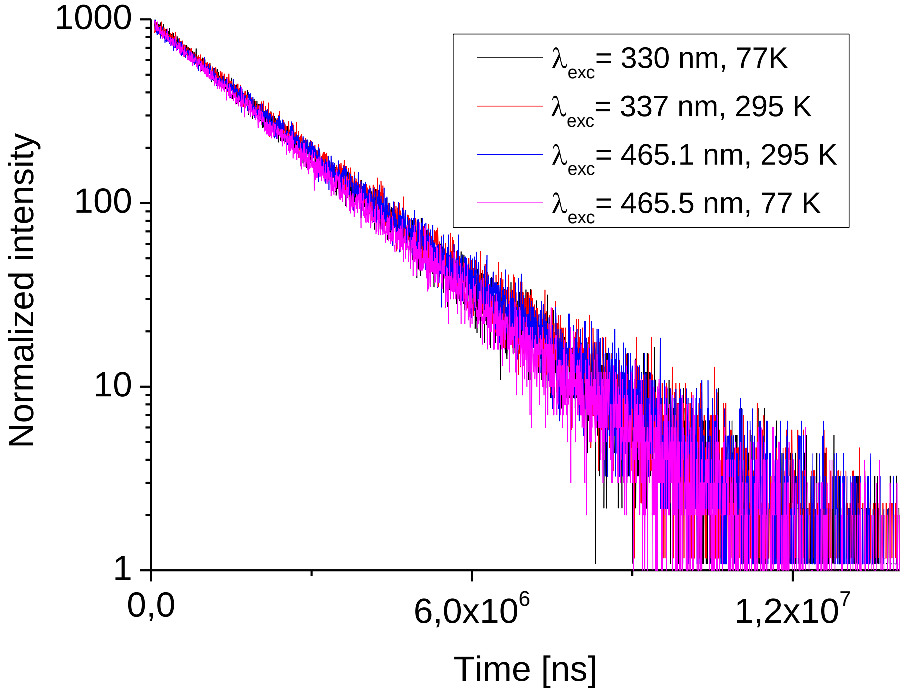

b)

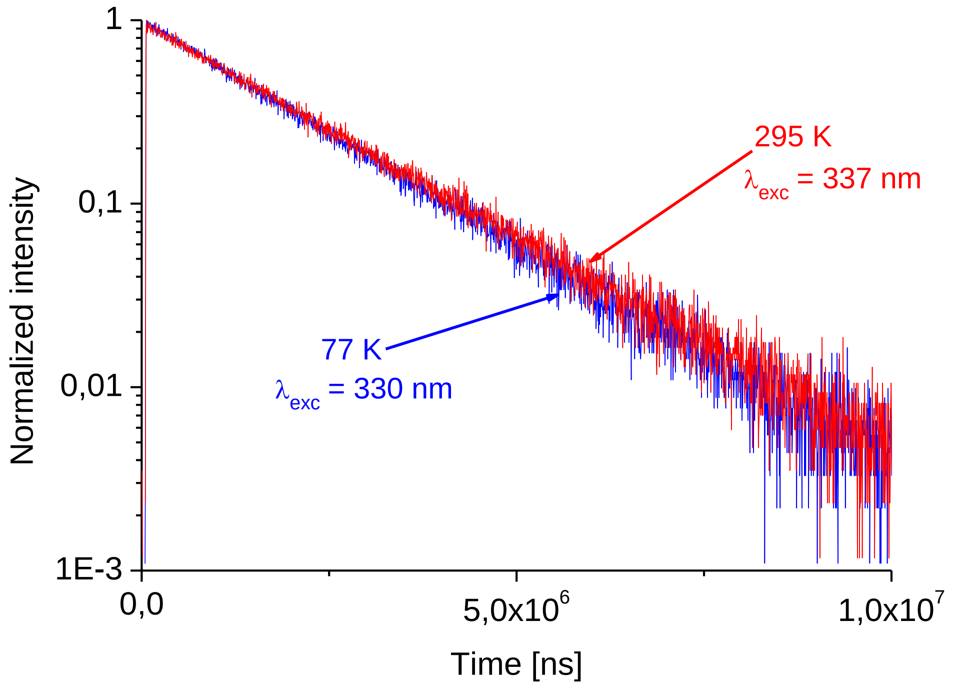

c)

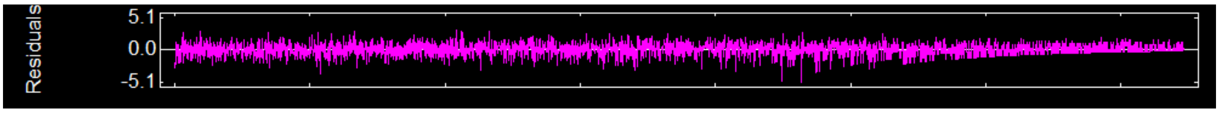

d)

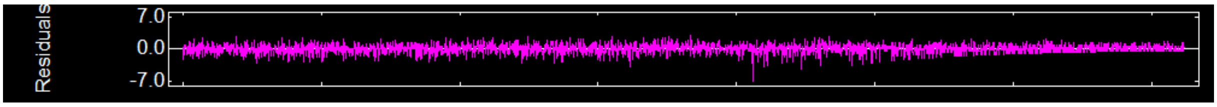

e)

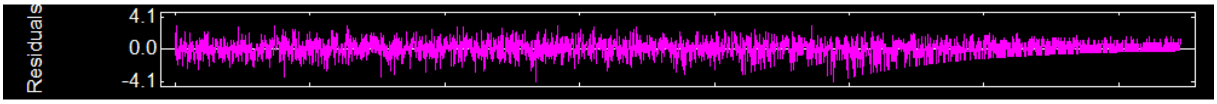

f)

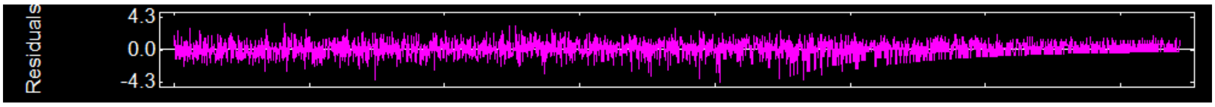

Supporting Figure S16 a), b) $^5D_0$ luminescence decay curves for **[$Eu_3L_3(\mu_2\text{-}F)_4(NO_3)_2$]($NO_3$)$_3$·2$H_2O$** monitored within the $^5D_0 \rightarrow ^7F_2$ transition, χ^2 = 1.009 (λ_{exc}=330 nm, 295 K), 1.003 (λ_{exc}=337 nm, 77 K), 1.077 (λ_{exc}=465.1 nm, 295 K), 1.001 (λ_{exc}=465.5 nm, 77 K) (χ^2 - the goodness of fit); c) °the residuals fit plot for λ_{exc}=330 nm and 295 K; d) the residuals fit plot for λ_{exc}=337 nm and 77 K; e) the residuals fit plot for λ_{exc}=465.1 nm and 295 K; f) the residuals fit plot for λ_{exc}=465.5 nm and 77 K.

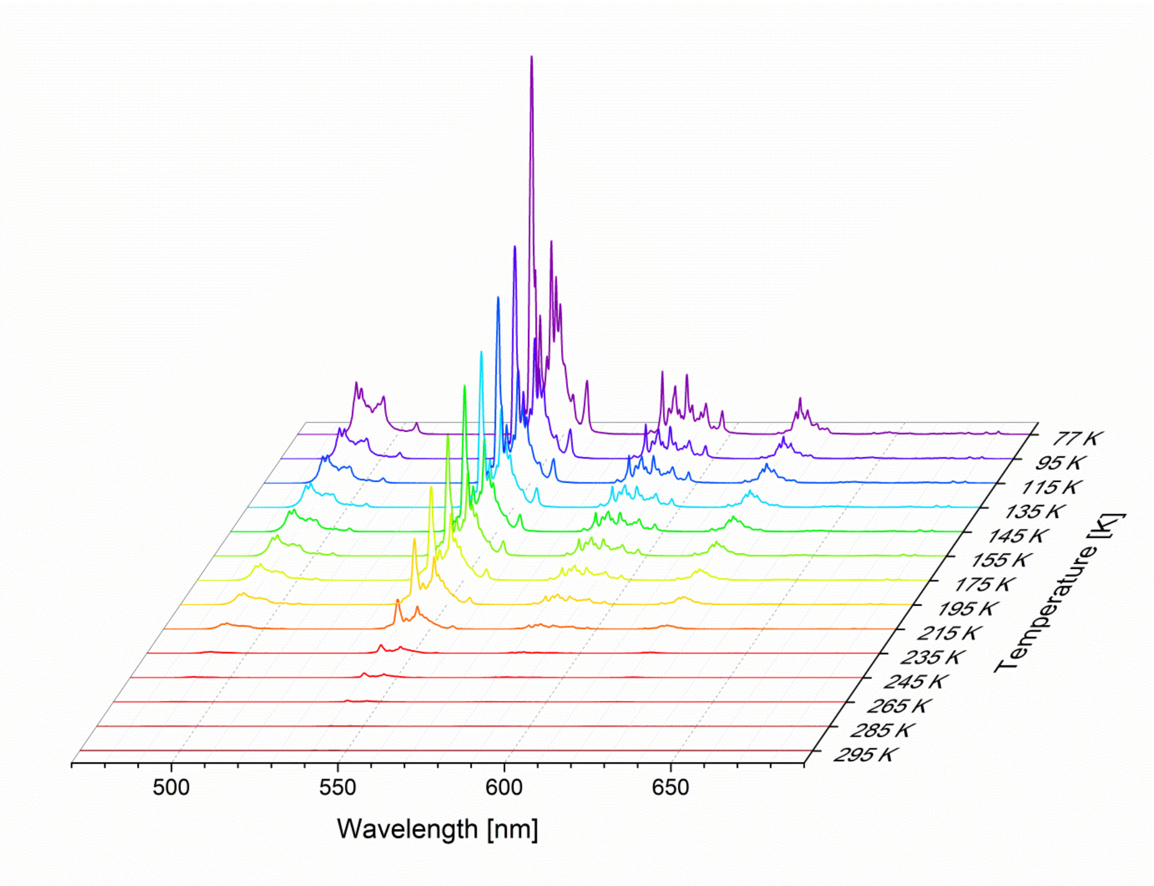

Supporting Figure S17. Emission spectra of **$[Tb_3L_3(\mu_2\text{-}F)_4(NO_3)_2](NO_3)_3\cdot 3H_2O$** in the solid state in the temperature range 295 - 77 K, λ_{exc} = 337 nm.

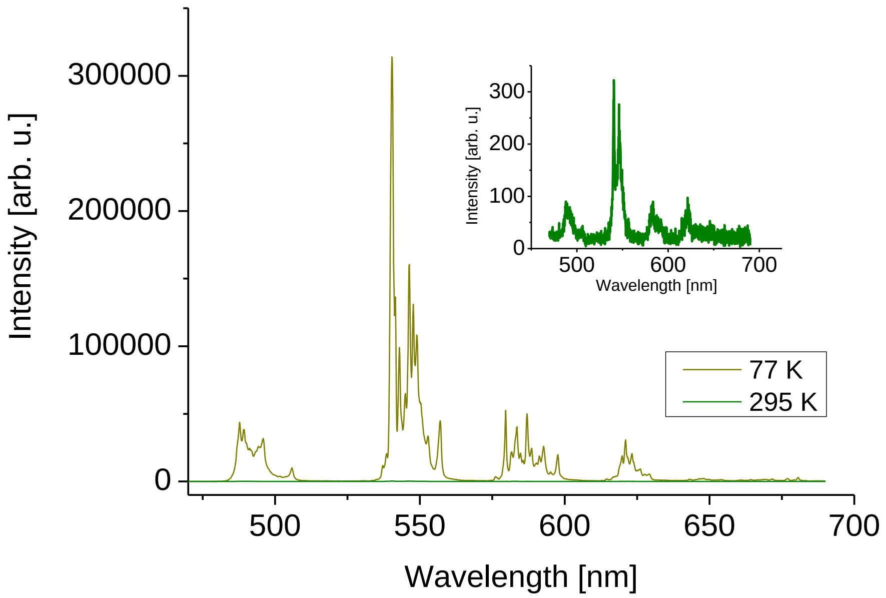

Supporting Figure S18. Comparison of $^5D_4$ emission intensity of **$[Tb_3L_3(\mu_2\text{-}F)_4(NO_3)_2](NO_3)_3\cdot 3H_2O$** at 295 and 77 K, λ_{exc} = 337 nm.

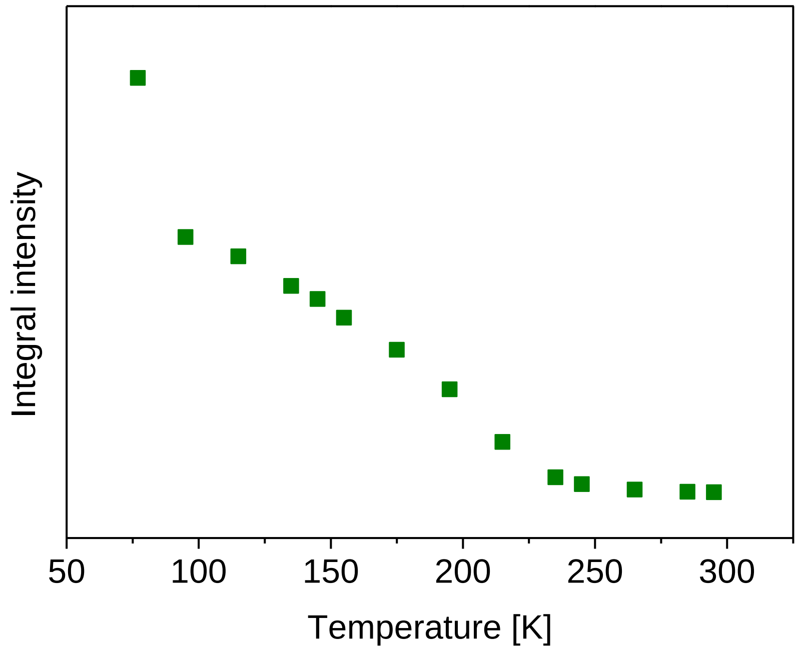

Supporting Figure S19. Temperature dependence of integral $^5D_4$ emission intensity for **[$Tb_3L_3(\mu_2$-F)$_4(NO_3)_2](NO_3)_3{\cdot}3H_2O$** measured in the range of $^5D_4 \rightarrow {}^7F_J$ (J = 6-0) transitions, λ_{exc} = 337 nm.

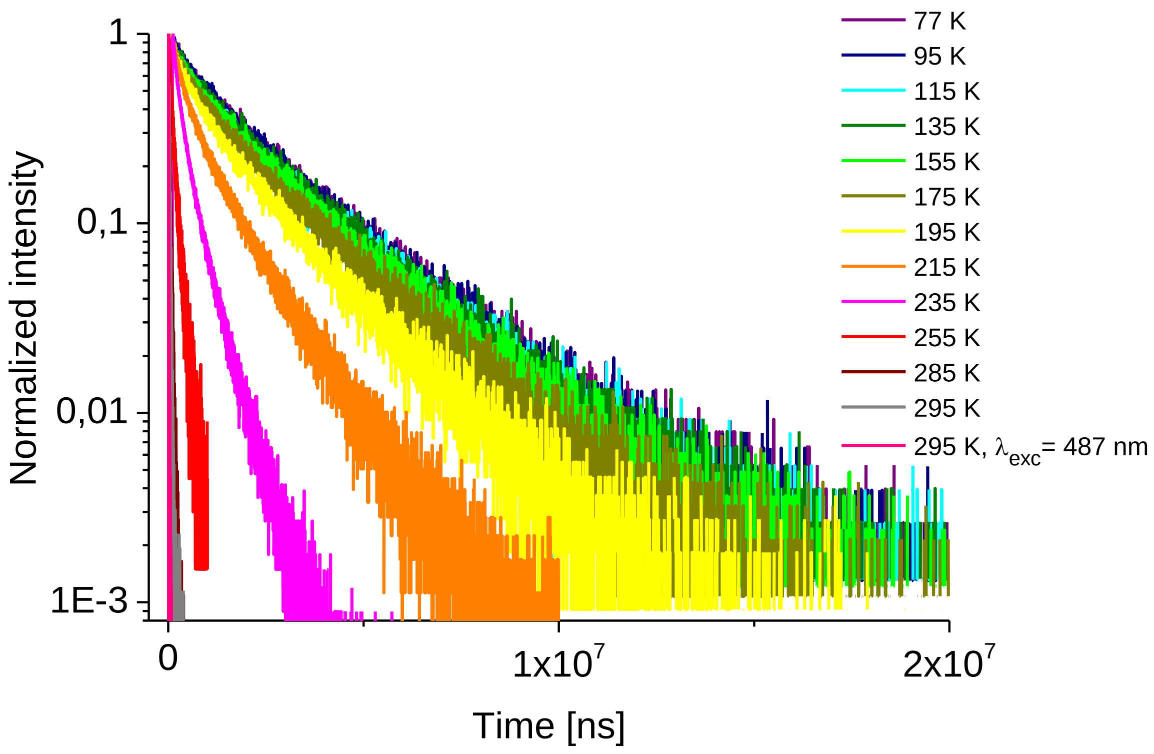

Supporting Figure S20. $^5D_4$ luminescence decay curves for **[$Tb_3L_3(\mu_2$-F)$_4(NO_3)_2](NO_3)_3{\cdot}3H_2O$** monitored within the $^5D_4 \rightarrow {}^7F_5$ transition, λ_{exc} = 337 nm.

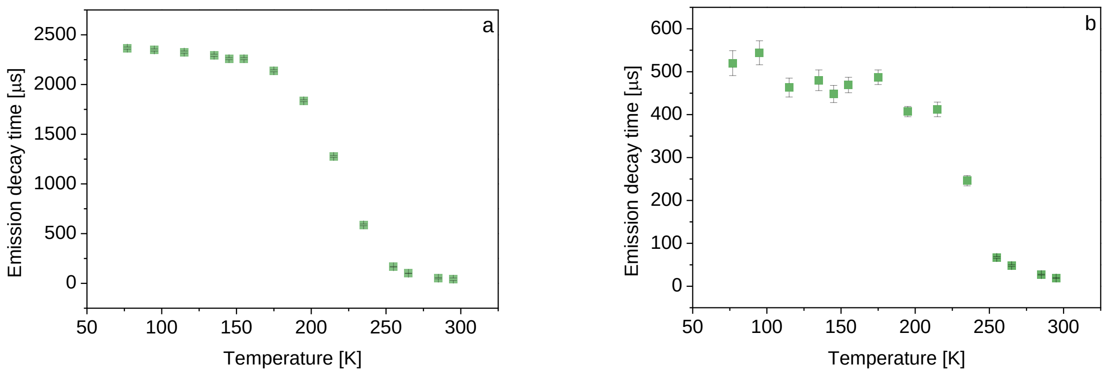

Supporting Figure S21. Temperature dependence of emission decay time for **[$Tb_3L_3(\mu_2$-$F)_4(NO_3)_2](NO_3)_3 \cdot 3H_2O$** monitored within the $^5D_4 \rightarrow ^7F_5$ transition with standard deviation of the decay time obtained by fitting the measurement data with a two-exponential function, λ_{exc} = 337 nm a) longer component of emission decay time, b) shorter component of emission decay time.

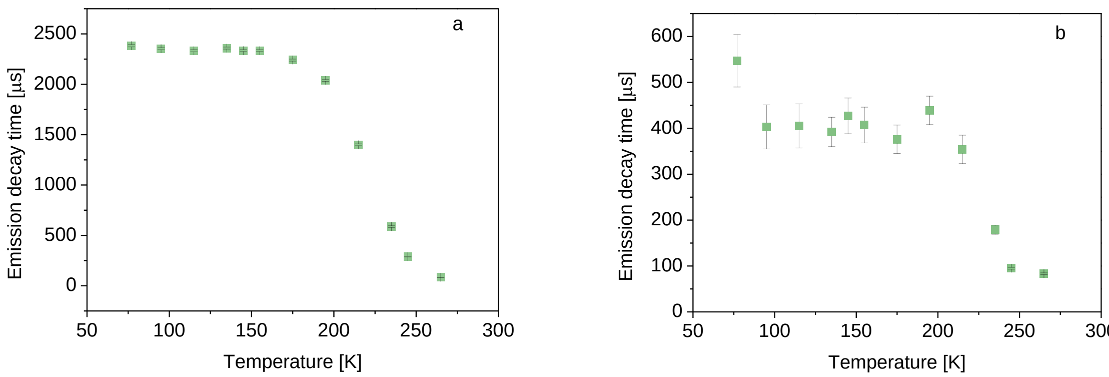

Supporting Figure S22. Temperature dependence of emission decay time for **[$Tb_3L_3(\mu_2$-$F)_4(NO_3)_2](NO_3)_3 \cdot 3H_2O$** monitored within the $^5D_4 \rightarrow ^7F_5$ transition with standard deviation of the decay time obtained by fitting the measurement data with a two-exponential function, λ_{exc} = 487.3 nm a) longer emission decay time, b) shorter emission decay time.

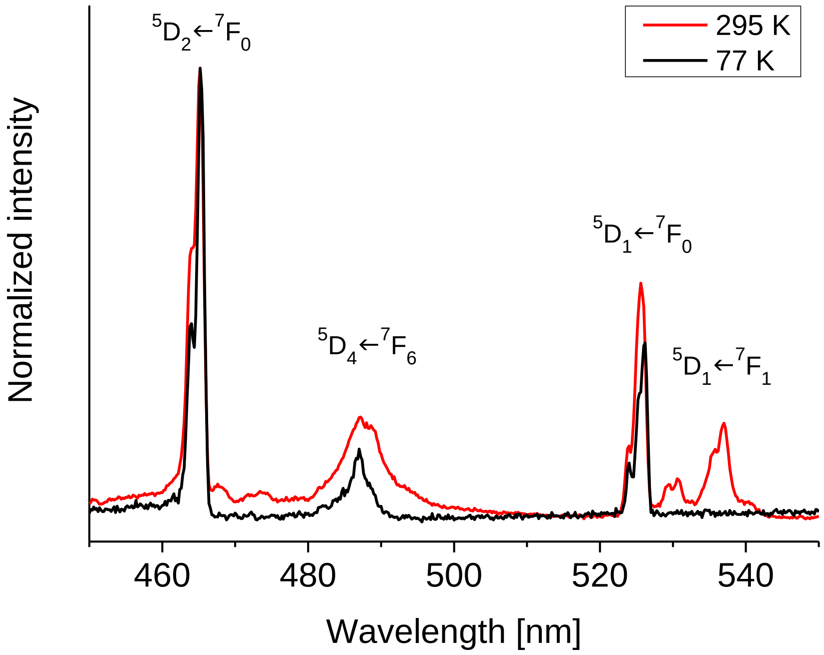

Supporting Figure S23. Excitation spectra in the range 450 - 550 nm, λ_{mon} = 702 nm.

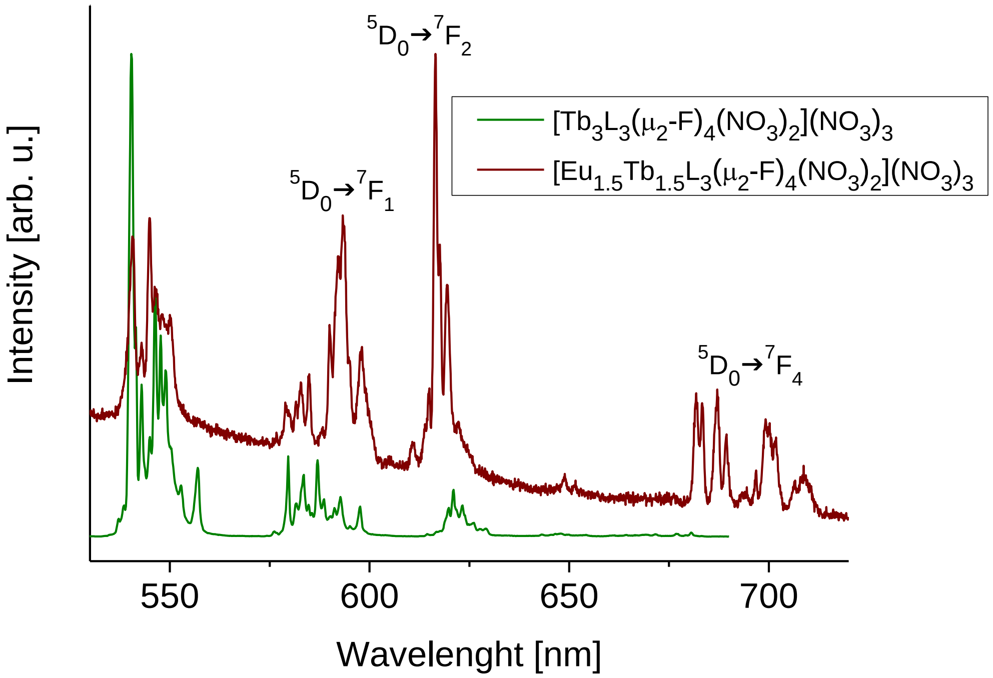

Supporting Figure S24. Emission spectra of **$[Tb_3L_3(\mu_2\text{-}F)_4(NO_3)_2](NO_3)_3\cdot3H_2O$** ($\lambda_{exc}$ = 337 nm)and **$[Eu_{1.5}Tb_{1.5}L_3(\mu_2\text{-}F)_4(NO_3)_2](NO_3)_3\cdot3H_2O$** (($\lambda_{exc}$ = 487 nm) at 77 K.

a)

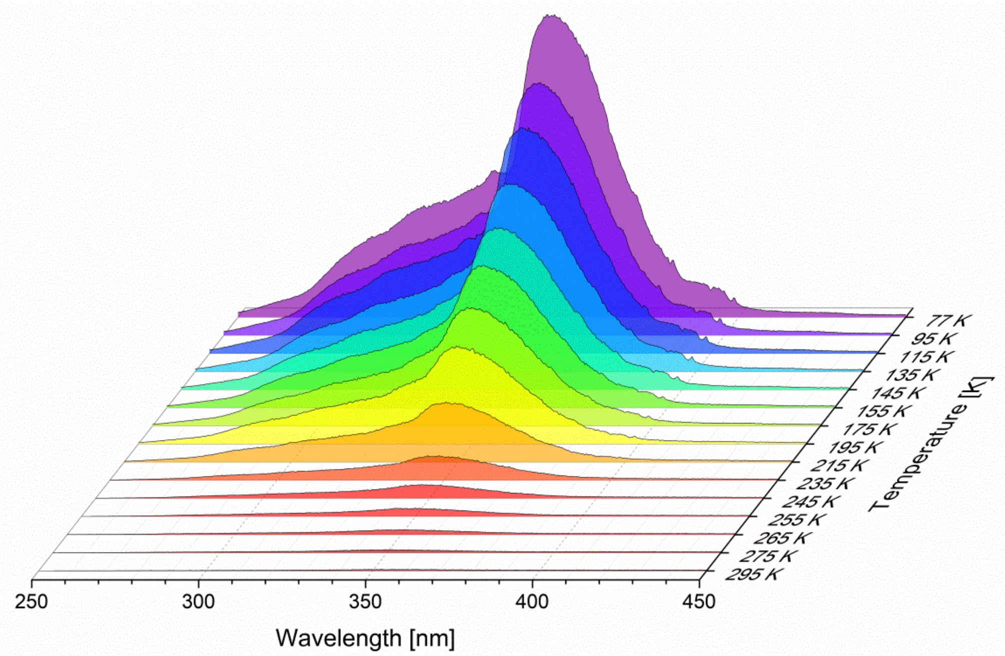

b)

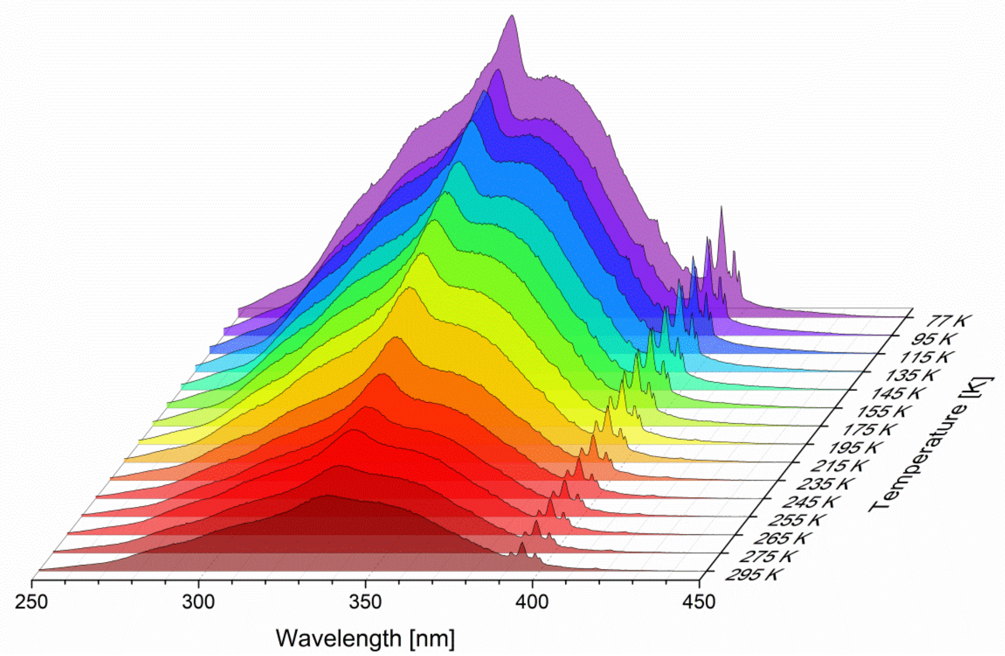

Supporting Figure S25. Excitation spectra of **$[Eu_{1.5}Tb_{1.5}L_3(\mu_2\text{-}F)_4(NO_3)_2](NO_3)_3\cdot 3H_2O$**, a) λ_{mon} = 540.5 nm, b) λ_{mon} = 616.5 nm.

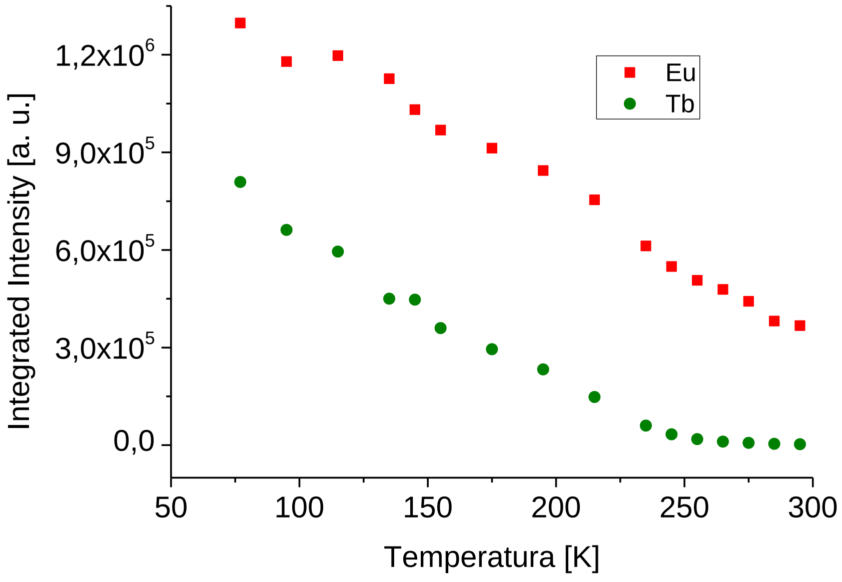

Supporting Figure S26. Dependence of the integral intensity of the bands corresponding to the $^5D_0 \rightarrow ^7F_2$ and $^5D_4 \rightarrow ^7F_5$ transitions on temperature for **[Eu$_{1.5}$Tb$_{1.5}$L$_3$(μ_2-F)$_4$(NO$_3$)$_2$](NO$_3$)$_3$·3H$_2$O** , λ_{exc} = 337 nm

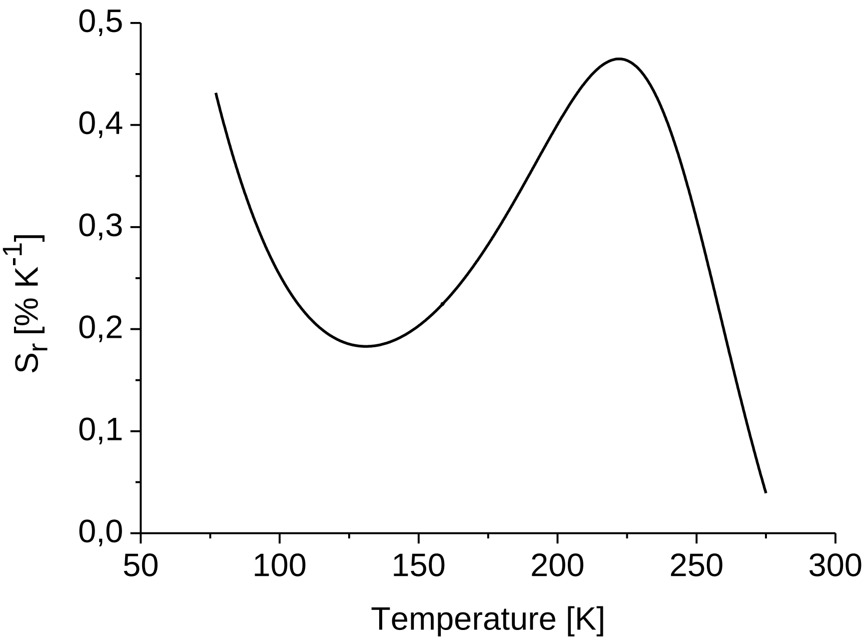

Supporting Figure S27. Relative thermal sensitivities for **[Eu$_{1.5}$Tb$_{1.5}$L$_3$(μ_2-F)$_4$(NO$_3$)$_2$](NO$_3$)$_3$·3H$_2$O**.

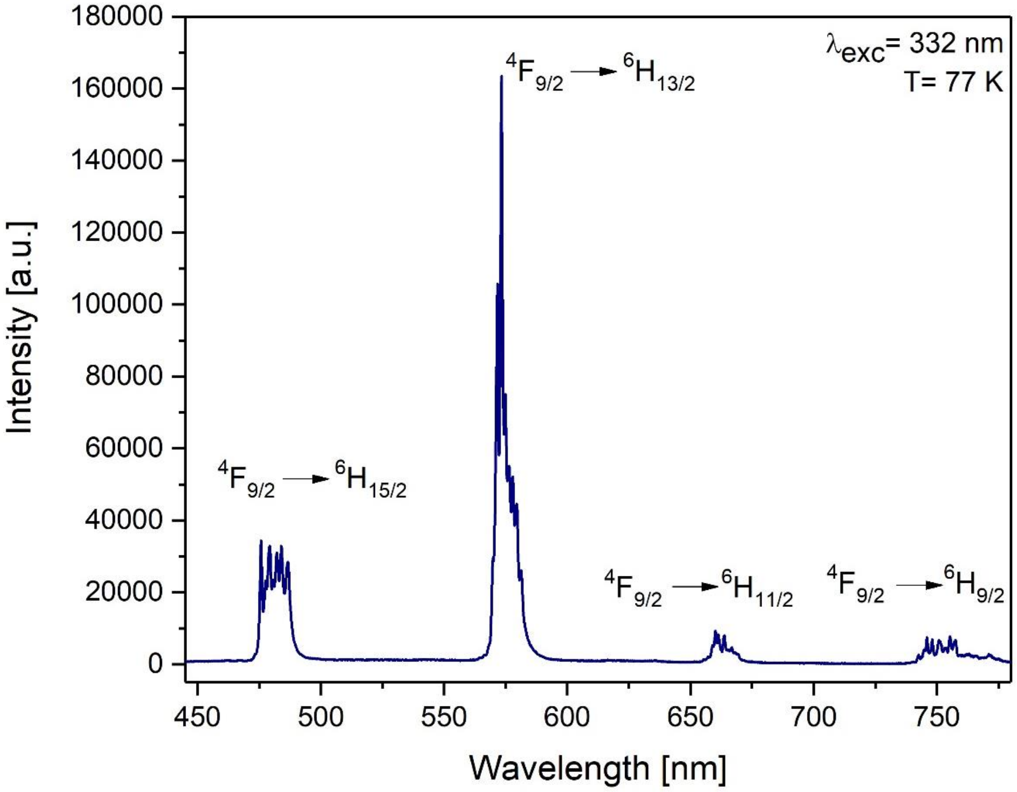

Supporting Figure S28. Emission spectrum of the mononuclear $[DyL(NO_3)_2](NO_3)$ coordination compound.

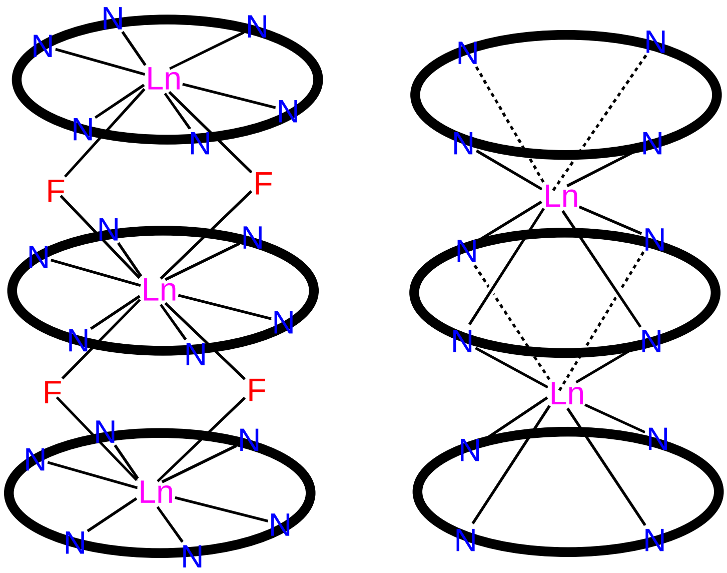

Supporting Figure S29. Comparison of the architecture of triple-decker lanthanide(III) complexes discussed in this work (left) with the architecture of triple-decker lanthanide(III) complexes based on phthalocyanines or porphyrins.

References

(1) Kasprzycka, E.; Trush, V. A.; Amirkhanov, V. M.; Jerzykiewicz, L; Malta, O. L.; Legendziewicz J.; Gawryszewska P. Contribution of energy transfer from the singlet state to the sensitization of Eu^{3+} and Tb^{3+} luminescence by sulfonylamidophosphates. *Chem Eur J.* **2017**;*23(6)*,1318-1330.

(2) Bunzli, J. C. G. On the design of highly luminescent lanthanide complexes. *Coordination Chemistry Reviews* **2015**, *293*, 19-47.

(3) Mooney, J.; Kambhampati, P. Get the basics right: Jacobian conversion of wavelength and energy scales for quantitative analysis of emission spectra. *J. Phys. Chem. Lett.* 2013, 4, 3316–3318, https://doi.org/10.1021/jz401508t.

(4) Carneiro Neto, A. N.; Teotonio, E. E. S.; de Sá, G. F.; Brito,H. F.; Legendziewicz, J.; Carlos, L. D.; Felinto, M. C. F. C.; Gawryszewska, P.; Moura, R. T.; Longo, R. L.; Faustino, W. M.;Malta, O. L. Modeling Intramolecular Energy Transfer in Lanthanide Chelates: A Critical Review and Recent Advances. In Handbook on the Physics and Chemistry of Rare

Earths; Bünzli, J.-C. G., Pecharsky, V. K., Eds.; Elsevier, **2019**; Vol. 56, Chapter 310, pp 55−162.

(5) C. D. S. Brites, A. Millán, L. D. Carlos, Lanthanides in Luminescent Thermometry. *Handb. Phys. Chem. Rare Earths* 2016, 49, 339.
